# COVID-19 Vaccination and Estimated Public Health Impact in California

**DOI:** 10.1001/jamanetworkopen.2022.8526

**Published:** 2022-04-22

**Authors:** Sophia T. Tan, Hailey J. Park, Isabel Rodríguez-Barraquer, George W. Rutherford, Kirsten Bibbins-Domingo, Robert Schechter, Nathan C. Lo

**Affiliations:** 1 Division of HIV, Infectious Diseases, and Global Medicine, University of California, San Francisco; 2 Department of Medicine, University of California, San Francisco; 3 Department of Epidemiology and Biostatistics, University of California, San Francisco; 4 Immunization Branch, California Department of Public Health, Richmond

## Abstract

**Importance:**

Despite widespread vaccination against COVID-19 in the United States, there are limited empirical data quantifying their public health impact in the population.

**Objective:**

To estimate the number of COVID-19 cases, hospitalizations, and deaths directly averted because of COVID-19 vaccination in California.

**Design, Setting, and Participants:**

This modeling study used person-level data provided by the California Department of Public Health (CDPH) on COVID-19 cases, hospitalizations, and deaths as well as COVID-19 vaccine administration from January 1, 2020, to October 16, 2021. A statistical model was used to estimate the number of COVID-19 cases that would have occurred in the vaccine era (November 29, 2020, to October 16, 2021) in the absence of vaccination based on the ratio of the number of cases among the unvaccinated (aged <12 years) and vaccine-eligible groups (aged ≥12 years) before vaccine introduction. Vaccine-averted COVID-19 cases were estimated by finding the difference between the projected and observed number of COVID-19 cases. Averted COVID-19 hospitalizations and deaths were assessed by applying estimated hospitalization and case fatality risks to estimates of vaccine-averted COVID-19 cases. As a sensitivity analysis, a second independent model was developed to estimate the number of vaccine-averted COVID-19 outcomes by applying published data on vaccine effectiveness to data on COVID-19 vaccine administration and estimated risk of COVID-19 over time.

**Exposure:**

COVID-19 vaccination.

**Main Outcomes and Measures:**

Number of COVID-19 cases, hospitalizations, and deaths estimated to have been averted because of COVID-19 vaccination.

**Results:**

There were 4 585 248 confirmed COVID-19 cases, 240 718 hospitalizations, and 70 406 deaths in California from January 1, 2020, to October 16, 2021, during which 27 164 680 vaccine-eligible individuals aged 12 years and older were reported to have received at least 1 dose of a COVID-19 vaccine in the vaccine era (79.5% of the eligible population). The primary model estimated that COVID-19 vaccination averted 1 523 500 (95% prediction interval [PI], 976 800-2 230 800) COVID-19 cases in California, corresponding to a 72% (95% PI, 53%-91%) relative reduction in cases because of vaccination. COVID-19 vaccination was estimated to have averted 72 930 (95% PI, 53 250-99 160) hospitalizations and 19 430 (95% PI, 14 840-26 230) deaths during the study period. The alternative model identified comparable findings.

**Conclusions and Relevance:**

This study provides evidence of the public health benefit of COVID-19 vaccination in the United States and further supports the urgency for continued vaccination.

## Key Points

**Question:**

How many COVID-19 cases, hospitalizations, and deaths were averted because of COVID-19 vaccination in California?

**Findings:**

In this modeling study using data from the California Department of Public Health, COVID-19 vaccination was estimated to have prevented more than 1.5 million COVID-19 cases, 72 000 hospitalizations, and 19 000 deaths during the first 10 months of vaccination, through October 16, 2021.

**Meaning:**

These findings suggest that COVID-19 vaccination had a large public health benefit in California, which can be generalized across the United States.

## Teaser

This decision analytical model estimates the number of COVID-19 cases, hospitalizations, and deaths averted because of COVID-19 vaccination in California.

## Introduction

COVID-19 has caused substantial morbidity, mortality, and socioeconomic disruption and disparity in the United States and globally. The COVID-19 vaccine has been a key tool for public health control of COVID-19, alongside public health measures for universal masking and social distancing.^1,2^ In the United States, implementation of COVID-19 vaccination took place in a phased approach, guided by recommendations from the Advisory Committee on Immunization Practices (ACIP) within the US Centers for Disease Control and Prevention. The ACIP recommendation prioritized vaccination based on risk of infection. Phase 1a of vaccination included health care personnel and residents of nursing facilities, phase 1b included frontline essential workers and adults 75 years or older, and phase 1c included adults 65 years or older and individuals with high-risk conditions. Phase 2 included vaccination of the general population 16 years or older.^3^ Initiation of phase 1a vaccination against COVID-19 began in December 2020,^3,4^ indicating the beginning of widespread vaccination in the United States.

Three vaccines are currently authorized in the United States: (1) BNT162b2 mRNA from Pfizer/BioNTech; (2) mRNA-1273 from Moderna; and (3) Ad26.COV2.S from Janssen. The landmark trials on these vaccines demonstrated high efficacy against clinical disease, hospitalization, and death. The efficacy of vaccine against symptomatic infection was 95%, 94%, and 66% for the BNT162b2, mRNA-1273, and Ad26.COV2.S vaccines, respectively, with even greater efficacy against hospitalization and death.^5,6,7^ Furthermore, published data on the effectiveness in the general population have demonstrated similar protection against clinical outcomes, with some waning for the BNT162b2 and Ad26.COV2.S vaccines^8^ and some differential vaccine effectiveness by SARS-CoV-2 variant.^9,10,11,12,13^ However, despite widespread implementation of COVID-19 vaccinations in the United States, there are limited data to estimate the overall population-level public health outcomes of vaccination regarding averted cases, hospitalization, and deaths from COVID-19.

This article reports on the estimated public health impact of COVID-19 vaccination by estimating the number of COVID-19 cases, hospitalizations, and deaths directly averted in the first 10 months of vaccination. This analysis uses the representative case example of California given the large population, geographic size, and epidemiologic variation within the state.

## Methods

We developed 2 independent statistical modeling approaches to estimate the number of averted COVID-19 cases because of direct effects of vaccination (ie, cases averted due to immunity) from the introduction of vaccine on November 29, 2020, to October 16, 2021. We applied estimated hospitalization and case fatality risks to estimate averted COVID-19 hospitalizations and deaths because of COVID-19 vaccination. In this analysis, we used person-level COVID-19 case data from the California Department of Public Health (CDPH) and public data on COVID-19 vaccination. The analytic code is available on GitHub.^14^ This project was approved by the institutional review board at the University of California, San Francisco. The requirement for informed consent was waived because the study relied on secondary data sets that were collected as part of public health surveillance. Study reporting followed the Consolidated Health and Economic Evaluation Reporting Standards (CHEERS) guidelines.^15^

### Data

We obtained deidentified person-level case data, including outcomes of hospitalization and death, for confirmed COVID-19 cases in California from January 1, 2020, to October 16, 2021, from CDPH. A case of COVID-19 was defined as a person whose positive SARS-CoV-2 molecular test was reported to the state, including both symptomatic cases and asymptomatic infections.^16^ COVID-19 hospitalizations and deaths were defined as individuals with confirmed COVID-19 cases who were hospitalized or died due to COVID-19 based on reporting to CDPH by health care practitioners. We excluded data on persons with missing age data (<1%).

We obtained publicly available CDPH vaccine administration data in 4 age groups (12-17 years, 18-49 years, 50-64 years, and ≥65 years) from July 27, 2020, to October 16, 2021.^17^ These age groupings were based on vaccine eligibility.^18^ We excluded vaccination in children aged 5 to 11 years old (vaccine coverage <0.01%) because they were ineligible during the study period.^19,20^ We additionally excluded vaccination that occurred before start of phase 1a of vaccination (<0.01%) or had missing age information (<0.02%). We aggregated both COVID-19 case and vaccination data from daily counts to weekly counts beginning January 1, 2020.

### Study Outcomes

The primary study outcomes were estimated COVID-19 cases, hospitalizations, and deaths averted because of the direct effects of COVID-19 vaccination. We estimated the relative reduction in COVID-19 cases because of vaccination, adjusting for vaccine coverage over time given initial periods of low vaccine coverage. We also provide alternative definitions for the relative reduction estimates (eAppendix in the Supplement). Secondary study outcomes include estimated averted COVID-19 outcomes and relative reduction in outcomes by age group. Study outcomes were chosen based on public health relevance.

### Statistical Analysis

We used 2 independent modeling approaches to estimate the number of averted COVID-19 cases because of vaccination in the vaccine era from November 29, 2020 to October 16, 2021. We defined the start of the vaccine era based on the approximate start of phase 1a of COVID-19 vaccination in California.^4^ We developed multiple estimation procedures that relied on different assumptions to improve the reliability and robustness of our study findings. All analyses were conducted in R version 3.6.0 (R Project for Statistical Computing).

#### Primary Statistical Model for COVID-19 Cases

In the primary model, we estimated the number of COVID-19 cases that would have otherwise occurred over time if vaccines were never available. This modeling approach used the unvaccinated population as a proxy for overall force of infection over time in the vaccine-eligible population. We defined our unvaccinated population as all children younger than 12 years, as they were ineligible for vaccination over the entire study period.

We used quasi-Poisson regression models to estimate the ratio between the log-transformed number of weekly COVID-19 cases in the unvaccinated population (<12 years) and the number of weekly cases in each of the 4 vaccine-eligible populations (aged 12-17 years, 18-49 years, 50-64 years, and ≥65 years) during the prevaccine era at the state level. The prevaccine era was defined as May 31 to November 28, 2020, to provide 6 months of data for model calibration. We fit separate models for each of the 4 age groups of the vaccine-eligible population.

We then applied each calibrated model to the observed COVID-19 cases in the unvaccinated population (<12 years) in the vaccine era (November 29, 2020, to October 16, 2021) to estimate the number of COVID-19 cases that would have occurred in each vaccine-eligible age group in the absence of vaccination. We computed the weekly difference between the projected number of COVID-19 cases in absence of vaccination with the observed number of COVID-19 cases from the CDPH data set (eAppendix in the Supplement). We summed the differences across age groups to estimate the total number of averted COVID-19 cases because of the direct protection of COVID-19 vaccination in California. We reported 95% prediction intervals (PIs) for study estimates. This model assumed that the relative risk of COVID-19 diagnosis remained constant over time between the unvaccinated population (<12 years) and vaccine-eligible age groups. This model did not explicitly account for person-level vaccination.

#### Alternative Statistical Model for COVID-19 Cases

In the second model, we used published data on vaccine effectiveness and estimated risk of COVID-19 in the vaccine era (November 29, 2020, to October 16, 2021) to estimate the number of COVID-19 cases averted because of the direct protection of vaccination. First, we estimated state-level weekly incidence of COVID-19 cases (defined as total cases per 100 000 susceptible persons) beginning January 1, 2020 (earliest available data for COVID-19 in California), in each of the 4 vaccine-eligible populations (aged 12-17 years, 18-49 years, 50-64 years, ≥65 years). We estimated the fraction of the population susceptible to infection over time by age group based on natural infection and/or vaccination. For natural immunity, we used data on reported COVID-19 cases and applied published estimates of age-specific clinical fractions to estimate the total number of infections (including subclinical infections) (Table 1; eAppendix in the Supplement).^6,7,8,22,23^ We made the simplifying assumption that natural infection provided perfect immunity, although we varied this in a sensitivity analysis. For vaccine-induced immunity, we used data on vaccine administration and published data on vaccine effectiveness.^5,6,7,8^ We estimated vaccine-induced immunity and waning specific to each of the COVID-19 vaccines (BNT162b2, mRNA-1273, and Ad26.COV2.S) over time (Table 1; eAppendix in the Supplement).

**Table 1.** Model Parameters for Alternative Model Estimation of Vaccine-Averted COVID-19 Cases

Parameter	Mean (95% CI)	Source
**Vaccine effectiveness by vaccine type**		
BNT162b2		
Between first and second dose	0.52 (0.3-0.68)	Polack et al,^6^ 2020
Weeks after second dose		
≤17	0.95 (0.90-0.98)	Polack et al,^6^ 2020
>17	0.77 (0.67-0.84)	Self et al,^8^ 2021
mRNA-1273		
Between first and second dose	0.82 (0.74-0.87)	Pilishvili et al,^21^ 2021
Weeks after second dose		
≤17	0.94 (0.89-0.97)	Baden et al,^5^ 2021
>17	0.92 (0.87-0.96)	Self et al,^8^ 2021
Ad26.COV2.S		
After first dose	0.66 (0.55-0.75)	Sadoff et al,^7^ 2021
**Subclinical fraction of SARS-CoV-2 infection by age group**		
<12	0.47 (0.32-0.62)	Sah et al,^22^ 2021
12-17	0.47 (0.32-0.62)	Sah et al,^22^ 2021
18-49 y		
18 y	0.47 (0.32-0.62)	Sah et al,^22^ 2021
19-49 y	0.32 (0.22-0.44)	Sah et al,^22^ 2021
50-64 y		
50-59 y	0.32 (0.22-0.44)	Sah et al,^22^ 2021
60-64 y	0.20 (0.13-0.29)	Sah et al,^22^ 2021
≥65	0.20 (0.13-0.29)	Sah et al,^22^ 2021

We applied the age-specific estimated risk of COVID-19 to the fraction of the corresponding age group susceptible to infection, removing vaccine-induced protection and thus simulating a scenario without vaccination. This estimation was conducted in the vaccine era (November 29, 2020, to October 16, 2021). We estimated 95% uncertainty intervals (UIs), which incorporated uncertainty in estimates of vaccine effectiveness over time and the age-specific clinical fraction generated through Monte Carlo simulation (eAppendix in the Supplement). This approach did not assume a fixed relationship in COVID-19 cases between the unvaccinated and vaccine-eligible groups. This model did assume complete reporting of both case and vaccination data and a constant clinical fraction among infections over time.

#### Statistical Analysis for Hospitalizations and Deaths

We estimated age-specific monthly risk of hospitalization and death among COVID-19 cases over time, as the severity of clinical outcome may have changed over time owing to clinical experience and COVID-19 directed therapies (eFigure 1 and eAppendix in the Supplement). We estimated averted COVID-19 hospitalizations and deaths by applying these hospitalizations and death risk estimates to vaccine-averted COVID-19 cases in the 3 vaccine-eligible age groups aged 18 years or older (18-49 years, 50-64 years, and ≥65 years). We also compared this estimate with published literature values in sensitivity analysis. We did not estimate these outcomes in younger age groups given their lower risk of hospitalization and death.

#### Sensitivity Analyses

We conducted several sensitivity analyses to evaluate varying assumptions and parameters in the model. We estimated the relative reduction in COVID-19 cases because of vaccination with alternative definitions, including no adjustment for vaccine coverage over time and considering different start dates for widespread vaccination based on age-specific vaccine eligibility (eAppendix in the Supplement).

In the primary model, we varied the calibration period and used other age group definitions for the unvaccinated population. We simulated changing risk of infection among children younger than 12 years from the Delta variant (eAppendix in the Supplement). In the alternative model, we varied vaccine effectiveness estimates to simulate the study findings under reduced vaccine effectiveness to the Delta variant and relaxed model assumptions of perfect immunity from natural infection to assess waning natural immunity on study outcomes (eAppendix in the Supplement). We ran the alternative model using literature estimates of vaccine effectiveness against hospitalization and death rather than estimates based on CDPH data (eAppendix in the Supplement).

## Results

### Descriptive Data

**Figure 1.**
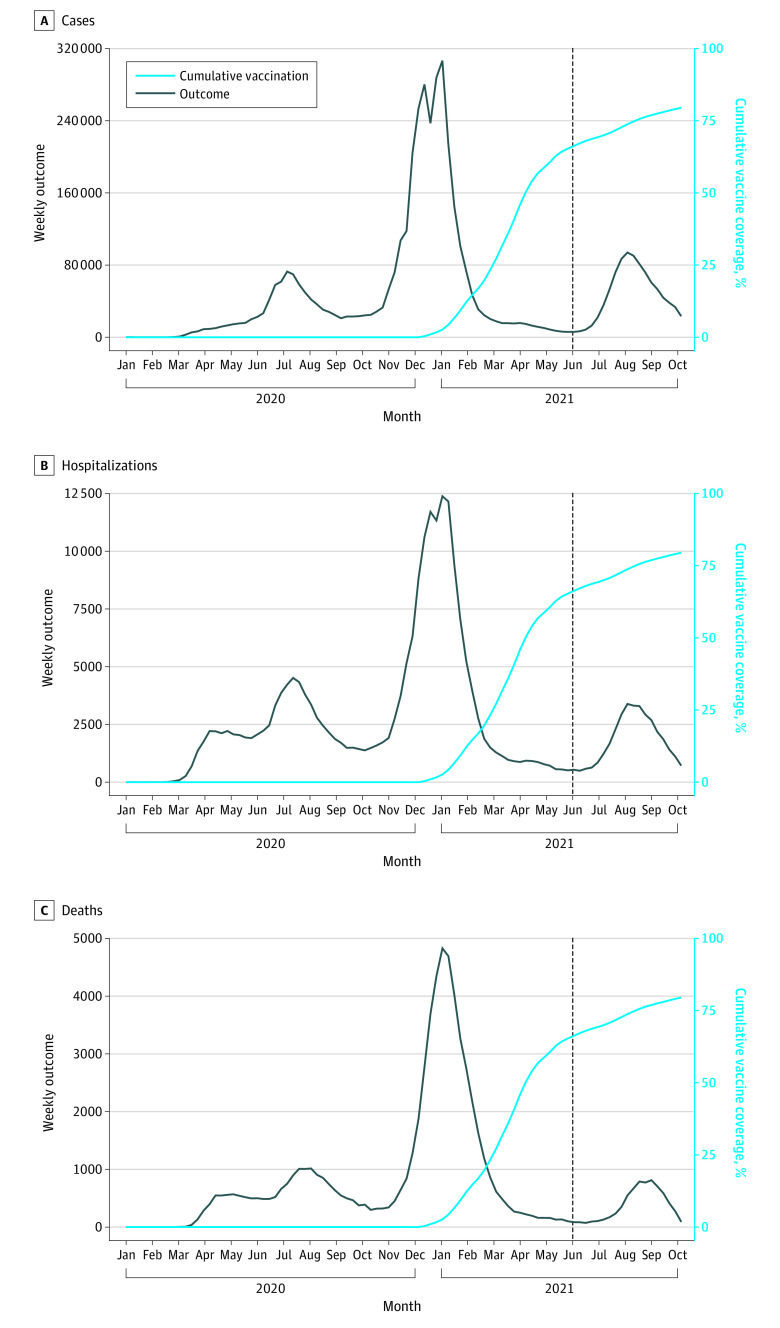
COVID-19 Cases, Hospitalizations, Deaths, and Vaccination Over Time in California Data on COVID-19 cases were obtained from the California Department of Public Health for the period of January 1, 2020, to October 16, 2021. Weekly absolute cases (A), hospitalizations (B), and deaths (C) were plotted (black line). Cumulative coverage of COVID-19 vaccination in the population aged 12 years or older was plotted (light blue line) using publicly available data from November 29, 2020, to October 16, 2021. Date of vaccination was defined as the date of first vaccine dose receipt in persons who received at least 1 dose of a COVID-19 vaccine (BNT162b2, mRNA-1273, or Ad26.COV2.S). The dashed line (black) represents the introduction of the Delta variant in California.

There were 4 588 146 reported COVID-19 cases in California from January 1, 2020, to October 16, 2021. We included 4 585 248 COVID-19 cases and excluded 2898 (0.06%) because of missing age data. A total of 3 276 260 COVID-19 cases occurred after November 28, 2020 (phase 1a of COVID-19 vaccination in California). There were 899 510 confirmed cases of COVID-19 after the Delta variant became a prominent circulating strain of SARS-CoV-2 in June 2021 (Figure 1).^24^

Among COVID-19 cases included in this analysis, there were 240 718 reported hospitalizations and 70 406 reported deaths (Figure 1). Both risk of hospitalization and risk of death varied over time and across age groups and was highest in the population aged 65 years or older (21.6% of reported cases resulted in hospitalization and 10.8% of reported cases resulted in death) (eFigure 1 in the Supplement). Overall estimates of the hospitalization and death risks (5% and 1.5%, respectively) were comparable with literature estimates (6% and 1%, respectively).^25^

Between November 29, 2020, and October 16, 2021, 27 164 680 persons aged 12 years and older (79.5%) were reported to have received at least 1 dose of a COVID-19 vaccine in California. We excluded 2022 individuals (<0.01%) who received vaccines before November 29, 2020 (Figure 1). Approximately 57% of vaccine-eligible individuals received the BNT162b2 vaccine, 36% received the mRNA-1273 vaccine, and 7% received the Ad26.COV2.S vaccine. We excluded 3924 individuals (<0.02%) with missing age from the vaccination data.

### COVID-19 Cases

#### Primary Model Results

**Figure 2.**
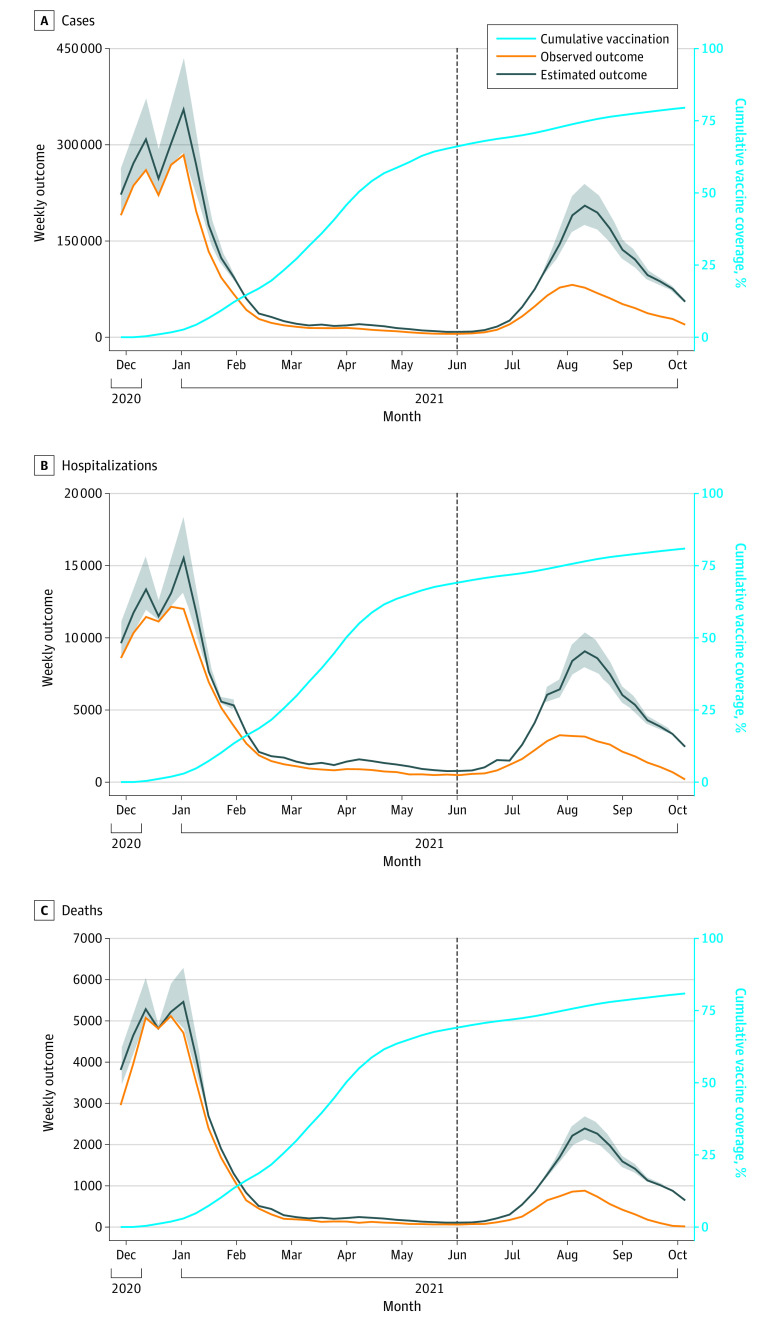
Primary Model Estimation of Averted COVID-19 Cases, Hospitalizations, and Deaths Because of COVID-19 Vaccination Observed COVID-19 cases in the population aged 12 years and older (A) and hospitalizations (B) and deaths (C) in the population aged 18 years and older was plotted (orange line) over time. Cumulative vaccine coverage of at least 1 dose of a COVID-19 vaccine was plotted over time in the vaccine-eligible population (light blue line). The ratio between cases in the unvaccinated group (aged <12 years) and each vaccine-eligible age group before phase 1a of vaccination was used to project COVID-19 cases in each vaccine-eligible age group in absence of vaccination (black line, with shaded areas indicating prediction intervals). We used weekly hospitalization and death risks to project hospitalizations and deaths in the absence of vaccination. The dashed line represents introduction of the Delta variant in California. The difference between estimated COVID-19 outcomes without vaccination (black line) and observed COVID-19 outcomes with vaccine (orange line) represents the averted COVID-19 outcomes because of vaccination.

We observed good model fit over the calibration period for the primary model in the prevaccine era (May 31 to November 28, 2020) (eFigure 2 in the Supplement). We estimated that 1 523 500 (95% PI, 976 800-2 230 800) COVID-19 cases were averted because of COVID-19 vaccination (Table 2 and Figure 2), which corresponded with a 72% (95% PI, 53%-91%) reduction in cases in the vaccine-eligible population after the start of phase 1a of vaccination (Table 2) and an 86% (95% PI, 81%-92%) reduction in cases when taking into account age-specific differences in vaccine eligibility (eTable 1 in the Supplement). The populations aged 12 to 17 years, 18 to 49 years, 50 to 64 years, and 65 years and older were estimated to have experienced 57%, 83%, 66%, and 49% reductions, respectively, in COVID-19 cases after the start of phase 1a of vaccination (Table 2; eFigures 3-6 in the Supplement), and these estimated reductions were greater when accounting for age-specific eligibility over time (eTable 1 in the Supplement). Approximately 1 005 500 (95% PI, 809 300-1 230 450) COVID-19 cases (66% of total cases averted) were estimated to have been averted after the Delta variant became the dominant strain of SARS-CoV-2 circulating in California in June 2021.^24^

**Table 2.** Comparison of Primary and Alternative Models Estimating Public Health Impact of COVID-19 Vaccination in California

Age group by model, y	Observed outcome, No.	Estimated outcome, No.	Averted outcome, No.	Relative reduction in outcome, %^a^	
				Unadjusted	Adjusted
**COVID-19 case**					
Primary model					
≥12	2 983 152	4 506 620 (95% PI, 3 959 910-5 213 980)	1 523 500 (95% PI, 976 800-2 230 800)	34 (95% PI, 25-43)	72 (95% PI, 53-91)
12-17	238 031	278 960 (95% PI, 268 300-290 120)	40 930 (95% PI, 30 300-52 100)	15 (95% PI, 11-18)	57 (95% PI, 44-70)
18-49	1 829 625	2 866 350 (95% PI, 2 444 720-3 412 980)	1 036 700 (95% PI, 615 100-1 588 400)	36 (95% PI, 25-46)	83 (95% PI, 58-100)
50-64	588 553	894 900 (95% PI, 810 040-1 003 720)	306 300 (95% PI, 221 500-415 200)	34 (95% PI, 27-41)	66 (95% PI, 52-79)
≥65	326 943	466 410 (95% PI, 436 850-507 170)	139 500 (95% PI, 109 900-180 200)	30 (95% PI, 25-36)	49 (95% PI, 42-59)
Alternative model					
≥12	2 983 152	4 385 300 (95% UI, 4 175 200-4 598 700)	1 402 100 (95% UI, 1 192 100-1 615 600)	32 (95% UI, 29-35)	68 (95% UI, 61-75)
12-17	238 031	316 790 (95% UI, 304 170-328 640)	78 760 (95% UI, 66 140-90 610)	25 (95% UI, 22-28)	97 (95% UI, 85-100)
18-49	1 829 625	2 640 320 (95% UI, 2 527 610-2 751 860)	810 700 (95% UI, 697 990-922 240)	31 (95% UI, 28-34)	71 (95% UI, 64-77)
50-64	588 553	909 830 (95% UI, 857 940-963 950)	321 280 (95% UI, 269 390-375 390)	35 (95% UI, 31-39)	68 (95% UI, 60-75)
≥65	326 943	518 340 (95% UI, 485 510-554 280)	191 390 (95% UI, 158 570-227 340)	37 (95% UI, 33-41)	61 (95% UI, 54-68)
**COVID-19 hospitalization**					
Primary model					
≥18	140 440	213 370 (95% PI, 193 690-239 590)	72 930 (95% PI, 53 250-99 150)	34 (95% PI, 27-41)	70 (95% PI, 56-84)
18-49	38 885	63 100 (95% PI, 54 410-74 540)	24 220 (95% PI, 15 520-35 650)	38 (95% PI, 29-48)	88 (95% PI, 66-100)
50-64	39 342	62 640 (95% PI, 56 940-69 960)	23 300 (95% PI, 17 600-30 620)	37 (95% PI, 31-44)	71 95% PI, (59-84)
≥65	62 213	87 630 (95% PI, 82 350-95 090)	25 410 (95% PI, 20 140-32 880)	29 (95% PI, 24-35)	48 (95% PI, 40-57)
Alternative model					
≥18	140 440	224 770 (95% UI, 212 200-237 950)	84 330 (95% UI, 71 760-97 510)	38 (95% UI, 34-41)	76 (95% UI, 69-83)
18-49	38 885	61 110 (95% UI, 58 270-63 910)	22 230 (95% UI, 19 390-25 030)	36 (95% UI, 33-39)	84 (95% UI, 77-90)
50-64	39 342	65 990 (95% UI, 62 000-70 140)	26 650 (95% UI, 22 660-30 790)	40 (95% UI, 37-44)	77 (95% UI, 70-84)
≥65	62 213	97 660 (95% UI, 91 930-103 900)	35 450 (95% UI, 29 710-41 690)	36 (95% UI, 32-40)	60 (95% UI, 53-66)
**COVID-19 death**					
Primary model					
≥18	45 060	64 490 (95% PI, 59 900-71 290)	19 430 (95% PI, 14 840-26 230)	30 (95% PI, 25-37)	61 (95% PI, 50-75)
18-49	3685	6420 (95% PI, 5560-7600)	2730 (95% PI, 1880-3920)	43 (95% PI, 34-52)	98 (95% PI, 78-100)
50-64	9803	15 870 (95% PI, 14 480,-17 720)	6070 (95% PI, 4670-7920)	38 (95% PI, 32-45)	73 (95% PI, 62-86)
≥65	31 572	42 200 (95% PI, 39 860-45 970)	10 630 (95% PI, 8290-14 400)	25 (95% PI, 21-31)	42 (95% PI, 34-52)
Alternative model					
≥18	45 060	67 680 (95% UI, 64 340-71 250)	22 620 (95% UI, 19 280-26 190)	33 (95% UI, 30-37)	68 (95% UI, 61-75)
18-49	3685	6100 (95% UI, 5810-6390)	2410 (95% UI, 2130-2700)	40 (95% UI, 37-42)	91 (95% UI, 84-97)
50-64	9803	16 290 (95% UI, 15 360-17 260)	6490 (95% UI, 5560-7460)	40 (95% UI, 36-43)	76 (95% UI, 69-83)
≥65	31 572	45 290 (95% UI, 43 170-47 590)	13 720 (95% UI, 11 600-16 020)	30 (95% UI, 27-34)	50 (95% UI, 44-56)

Abbreviations: PI, prediction interval; UI, uncertainty interval.

^a^ Relative reduction in outcomes were adjusted for the mean vaccine coverage in the population during the vaccine era, while the unadjusted estimate did not account for vaccine coverage.

We performed sensitivity analyses on the primary model, including varying date to define vaccine introduction (eTable 3 in the Supplement), testing alternative age definitions for the vaccine-eligible population (eTable 2 in the Supplement), and changing transmission dynamics due to the Delta variant (eTable 4 in the Supplement). The findings of these sensitivity analyses were overall similar to the main results.

#### Alternative Model Results

**Figure 3.**
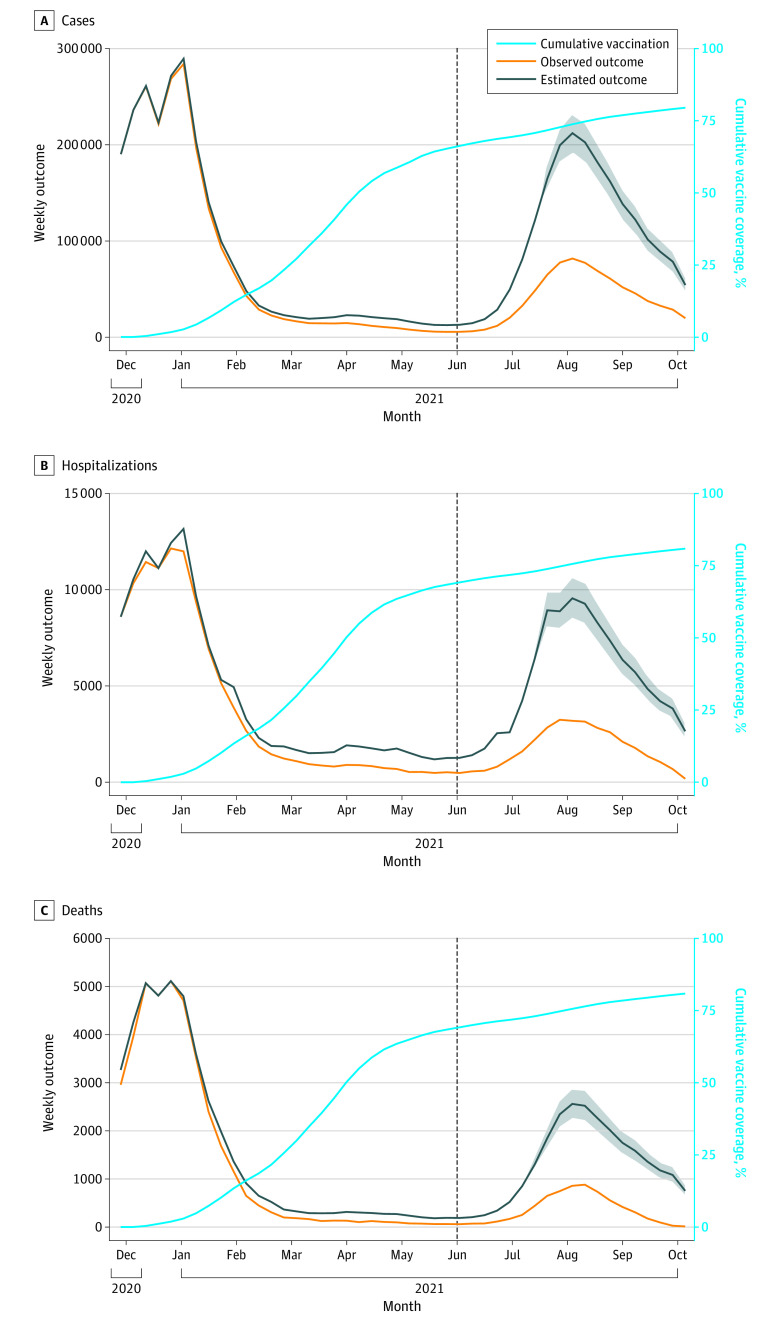
Alternative Model Estimation of Averted COVID-19 Cases, Hospitalizations, and Deaths Because of COVID-19 Vaccination Observed COVID-19 cases in the population aged 12 years and older (A) and hospitalizations (B) and deaths (C) in the population aged 18 years and older were plotted (orange line). Cumulative vaccine coverage of at least 1 dose of a COVID-19 vaccine was plotted (light blue line). This analysis used an alternative modeling approach for estimating vaccine-averted COVID-19 cases. COVID-19 case incidence was estimated over time, accounting for natural and vaccine-induced immunity. This COVID-19 incidence was used to project weekly cases in the vaccine-eligible group, under the scenario of no vaccination (black line, with shaded areas indicating uncertainty intervals). Monthly COVID-19 hospitalization and death risks were applied to estimate COVID-19 hospitalizations and deaths. The difference between estimated COVID-19 outcomes without vaccination (black line) and observed COVID-19 outcomes with vaccine (orange line) represents the estimated averted COVID-19 outcomes because of vaccination. The dashed line represents introduction of the Delta variant in California.

In the alternative modeling approach, we estimated that 1 402 100 (95% UI, 1 192 100-1 615 600) cases were averted because of COVID-19 vaccination from November 29, 2020, to October 16, 2021 (Table 2 and Figure 3). We estimated that vaccination contributed to a 68% (95% UI, 61%-75%) reduction in cases in the population aged 12 years or older after the start of vaccination when adjusting for vaccine coverage (Table 2) and 93% (95% UI, 86%-99%) reduction in cases when accounting for age-specific differences in vaccine eligibility (eTable 1 in the Supplement). The populations aged 12 to 17 years, 18 to 49 years, 50 to 64 years, and 65 years or older were estimated to have experienced 97%, 71%, 68%, and 61%, respectively, reductions in COVID-19 cases after the start of phase 1a of vaccination (Table 2; eFigures 3-6 in the Supplement), and these estimated reductions in cases were greater when accounting for differences in vaccine eligibility (eTable 1 in the Supplement). We estimated that 90% of averted cases were prevented after the introduction of the Delta variant.

In sensitivity analysis, we obtained comparable results when accounting for the possibility of reduced vaccine effectiveness against the Delta variant; we estimated that 1 106 300 (95% UI, 913 300-1 308 650) cases were prevented, and there was an estimated 58% (95% UI, 50%-65%) reduction in cases (eTable 5 in the Supplement). In this sensitivity analysis, 87% of estimated averted COVID-19 cases were prevented during widespread transmission of the Delta variant. We additionally found similar results when relaxing the assumption that natural infection provided full immunity (eTable 6 in the Supplement).

### COVID-19 Hospitalizations and Deaths

Using estimates of averted COVID-19 cases from the primary model, we estimated that 72 930 (95% PI, 53 250-99 150) hospitalizations and 19 430 (95% PI, 14 840-26 230) deaths were averted in the population aged 18 years or older because of COVID-19 vaccination (Table 2 and Figure 2). From the alternative model of cases, we estimated there were 84 330 (95% UI, 71 760-97 510) hospitalizations and 22 620 (95% UI, 19 280-26 190) deaths prevented because of vaccination (Table 2 and Figure 3). We found similar results for both hospitalizations and deaths across all sensitivity analyses (eTables 1-8 in the Supplement).

## Discussion

In this study, we estimated that COVID-19 vaccination had a large public health benefit by averting COVID-19 cases and related hospitalizations and deaths, which likely generalizes across the United States. More than 1.5 million COVID-19 cases were estimated to have been prevented because of protection by COVID-19 vaccination programs during the first 10 months of widespread vaccination in California. We additionally estimated that there were more than 72 000 vaccine-averted hospitalizations and at least 19 000 vaccine-averted deaths. Vaccination contributed to an estimated greater than 70% reduction in cases. These study findings are strengthened by our second modeling approach, which had comparable findings despite relying on distinct assumptions. Furthermore, our study likely provides a lower bound for the estimated public health impact of COVID-19 vaccination given we only estimated the direct effects of vaccination.

Our findings suggest that COVID-19 vaccination had especially invaluable benefit for mitigating the surge of COVID-19 cases due to the Delta variant, which is more infectious than other previously identified variants of SARS-CoV-2 during the first 2 years of the pandemic.^23^ A large number of COVID-19 outcomes were estimated to have been prevented after the introduction of the SARS-CoV-2 Delta variant in California. The primary model and alternative model estimated that more than 65% to 90% of averted COVID-19 cases occurred after the Delta variant became the prominent variant of SARS-CoV-2 circulating in California, with similar estimates of averted hospitalizations and deaths. The burden of COVID-19 cases and more severe outcomes would have been significantly greater in the absence of vaccination (Figure 2 and Figure 3), including in the Omicron surge. We found similar estimates for vaccination in averting severe clinical outcomes when additionally considering increased infectiousness of the Delta variant in the primary model and reduced effectiveness of vaccines against the Delta variant in the alternative model.

We estimated a 72% reduction in COVID-19 cases in the vaccine-eligible population in California from the start of phase 1a of vaccination when adjusting for the average vaccine coverage. This provided a conservative estimate of the true reduction given that vaccine eligibility across age groups varied over time. Other factors associated with this estimate not being larger include imperfect vaccine effectiveness and waning immunity. When accounting for differences in vaccine eligibility by age, we found an estimated 86% reduction in COVID-19 cases. However, this still likely represents an underestimate, as early vaccine eligibility was determined by occupational and health risk, which our data was unable to fully capture. Other model-based analyses on the population-level outcomes of vaccination have been previously undertaken, and in comparison, support the large public health benefit of COVID-19 vaccination.^26^

A key strength of the analysis is the development of 2 distinct modeling approaches to improve reliability of the study findings. While each analysis of estimating averted COVID-19 cases relied on limiting assumptions, the assumptions were nonoverlapping, and overall findings for all study outcomes were similar. Notably, the primary modeling approach estimated a greater number of outcomes averted early in the vaccine era. The alternative modeling approach estimated greater vaccine benefit after the widespread circulation of the Delta variant (Figure 2 and Figure 3). The alternative modeling approach estimated more cases averted in the populations aged 12 to 17 years and 65 years or older than the primary modeling approach and more hospitalizations and deaths averted in the populations aged 50 to 64 years and 65 years or older (Table 2). The PIs from the primary modeling approach include larger uncertainty than the UIs of the alternative modeling approach, although these intervals represent different statistical entities and are broadly overlapping.

### Limitations

Our study has limitations. We report predominately on the estimated direct effects of vaccination and were not able to capture the indirect effects of vaccination (ie, averted outcomes due to reduced SARS-CoV-2 transmission), meaning the overall benefits of vaccination, which includes both direct and indirect effects of vaccination, is likely much larger than estimated.

Our primary modeling approach for cases relied on the key assumption that COVID-19 cases in the unvaccinated population (aged <12 years) remained a robust indicator of cases in each vaccine-eligible group over time. We assumed that the relative risk of SARS-CoV-2 infection between the vaccine-ineligible (aged <12 years) and vaccine-eligible (aged ≥12 years) populations was stable over time, as well as stable testing practices within and between these age groups. Relative risk and testing practices likely changed over time and differentially between groups, especially as children returned to schools and had access to increased SARS-CoV-2 testing in the fall of 2021.^27^ To address this, we developed the alternative modeling approach that did not make this assumption and ultimately had similar study findings.

Our alternative modeling approach used the susceptibility profile of the vaccine-eligible population over time to estimate the COVID-19 cases prevented by vaccination. The key assumption in the alternative model was complete reporting of COVID-19 cases and vaccination, although this assumption was relaxed for estimating the relative reduction outcome. Similarly, we assumed the symptomatic fraction of reported SARS-CoV-2 infections remained constant over time, even with the introduction of vaccines (eAppendix in the Supplement). An increase in asymptomatic screening over time would bias the study to overestimate the public health benefit of vaccination, while an increase in the asymptomatic fraction of infection over time due to vaccine effectiveness would bias the study to underestimate the public health impact of vaccination.

Our approach to estimating hospitalizations and deaths prevented by COVID-19 vaccination has limitations. We were unable to estimate age-specific risks of hospitalization and death among cases solely in the unvaccinated population. Our estimates of the risk of severe outcomes reflect overall risk of hospitalization and death among both vaccinated and unvaccinated individuals in the vaccine-eligible population, which suggests that our results likely underestimate the direct effects of vaccination on severe COVID-19 outcomes, although these estimates were similar to published values elsewhere.

## Conclusions

In this study, our estimates suggest that COVID-19 vaccination had a substantial public health benefit by reducing COVID-19 cases, hospitalizations, and deaths in California, especially during widespread transmission of the Delta variant. The value of vaccination is likely to be larger with the emergence of more transmissible variants, such as the Omicron variant. This study provides evidence on the public health benefit of COVID-19 vaccination in the United States and further supports the urgency for continued vaccination.

## Notes

Supplement. **eAppendix. **Supplemental Methods **eFigure 1. **Monthly Risk of Hospitalization and Death Among COVID-19 Cases **eFigure 2.** Model Calibration of Primary Model for Ratio Between COVID-19 Cases in the Unvaccinated and Vaccine-Eligible Age Groups in the Prevaccine Era **eFigure 3. **Comparison of Primary and Alternative Models of Estimating Vaccine-Averted Outcomes in the Population Aged 12 to 17 Years **eFigure 4. **Comparison of Primary and Alternative Models of Estimating Vaccine-Averted Outcomes in the Population Aged 18 to 49 Years **eFigure 5. **Comparison of Primary and Alternative Models of Estimating Vaccine-Averted Outcomes in the Population Aged 50 to 64 Years **eFigure 6. **Comparison of Primary and Alternative Models of Estimating Vaccine-Averted Outcomes in the Population Aged 65 Years or Older **eTable 1.** Sensitivity Analysis on Association of Age-Specific Vaccine Eligibility With Vaccine-Averted Outcomes in California **eTable 2. **Sensitivity Analysis of the Primary Model Measuring Averted COVID-19 Outcomes When Varying the Start of the Vaccine Era in California **eTable 3. **Sensitivity Analysis of the Primary Model Measuring Averted COVID-19 Outcomes in Vaccine-Eligible Population Using Different Unvaccinated Age Groups **eTable 4. **Sensitivity Analysis of the Primary Model Measuring Changing Risk of Infection From the Delta Variant in Unvaccinated Population **eTable 5. **Sensitivity Analysis of the Alternative Modeling Adjusting for Possible Reduced Vaccine Effectiveness Against the SARS-CoV-2 Delta Variant **eTable 6. **Sensitivity Analysis of the Alternative Modeling With Waning Natural Immunity **eTable 7. **Sensitivity Analysis Estimating Averted Hospitalizations and Deaths Using Literature Estimates of Risk of Hospitalization and Death **eTable 8. **Sensitivity Analysis Estimating Averted Hospitalizations and Deaths Using Estimates of Vaccine Effectiveness Against Hospitalization and Death
eReferences.
