# Supplementary material for: COVID-19 Vaccination and Estimated Public Health Impact in California
Source: JAMA Netw Open. 2022 Apr 22;5(4):e228526. doi: 10.1001/jamanetworkopen.2022.8526 (PMC9034409; doi:10.1001/jamanetworkopen.2022.8526)
Supplement: Supplement. — eAppendix. Supplemental Methods eFigure 1. Monthly Risk of Hospitalization and Death Among COVID-19 Cases eFigure 2. Model Calibration of Primary Model for Ratio Between COVID-19 Cases in the Unvaccinated and Vaccine-Eligible Age Groups in the Prevaccine Era eFigure 3. Comparison of Primary and Alternative Models of Estimating Vaccine-Averted Outcomes in the Population Aged 12 to 17 Years eFigure 4. Comparison of Primary and Alternative Models of Estimating Vaccine-Averted Outcomes in the Population Aged 18 to 49 Years eFigure 5. Comparison of Primary and Alternative Models of Estimating Vaccine-Averted Outcomes in the Population Aged 50 to 64 Years eFigure 6. Comparison of Primary and Alternative Models of Estimating Vaccine-Averted Outcomes in the Population Aged 65 Years or Older eTable 1. Sensitivity Analysis on Association of Age-Specific Vaccine Eligibility With Vaccine-Averted Outcomes in California eTable 2. Sensitivity Analysis of the Primary Model Measuring Averted COVID-19 Outcomes When Varying the Start of the Vaccine Era in California eTable 3. Sensitivity Analysis of the Primary Model Measuring Averted COVID-19 Outcomes in Vaccine-Eligible Population Using Different Unvaccinated Age Groups eTable 4. Sensitivity Analysis of the Primary Model Measuring Changing Risk of Infection From the Delta Variant in Unvaccinated Population eTable 5. Sensitivity Analysis of the Alternative Modeling Adjusting for Possible Reduced Vaccine Effectiveness Against the SARS-CoV-2 Delta Variant eTable 6. Sensitivity Analysis of the Alternative Modeling With Waning Natural Immunity eTable 7. Sensitivity Analysis Estimating Averted Hospitalizations and Deaths Using Literature Estimates of Risk of Hospitalization and Death eTable 8. Sensitivity Analysis Estimating Averted Hospitalizations and Deaths Using Estimates of Vaccine Effectiveness Against Hospitalization and Death eReferences. [file jamanetwopen-e228526-s001.pdf]

Supplemental Online Content

Tan ST, Park HJ, Rodríguez-Barraquer I, et al. COVID-19 vaccination and estimated public health impact in California. *JAMA Netw Open.* 2022;5(4):e228526. doi:10.1001/jamanetworkopen.2022.8526

eAppendix. Supplemental Methods

eFigure 1. Monthly Risk of Hospitalization and Death Among COVID-19 Cases

eFigure 2. Model Calibration of Primary Model for Ratio Between COVID-19 Cases in the Unvaccinated and Vaccine-Eligible Age Groups in the Prevaccine Era

eFigure 3. Comparison of Primary and Alternative Models of Estimating Vaccine-Averted Outcomes in the Population Aged 12 to 17 Years

eFigure 4. Comparison of Primary and Alternative Models of Estimating Vaccine-Averted Outcomes in the Population Aged 18 to 49 Years

eFigure 5. Comparison of Primary and Alternative Models of Estimating Vaccine-Averted Outcomes in the Population Aged 50 to 64 Years

eFigure 6. Comparison of Primary and Alternative Models of Estimating Vaccine-Averted Outcomes in the Population Aged 65 Years and Older

eTable 1. Sensitivity Analysis on Association of Age-Specific Vaccine Eligibility With Vaccine-Averted Outcomes in California

eTable 2. Sensitivity Analysis of the Primary Model Measuring Averted COVID-19 Outcomes When Varying the Start of the Vaccine Era in California

eTable 3. Sensitivity Analysis of the Primary Model Measuring Averted COVID-19 Outcomes in Vaccine-Eligible Population Using Different Unvaccinated Age Groups

eTable 4. Sensitivity Analysis of the Primary Model Measuring Changing Risk of Infection From the Delta Variant in Unvaccinated Population

eTable 5. Sensitivity Analysis of the Alternative Modeling Adjusting for Possible Reduced Vaccine Effectiveness Against the SARS-CoV-2 Delta Variant

eTable 6. Sensitivity Analysis of the Alternative Modeling With Waning Natural Immunity

eTable 7. Sensitivity Analysis Estimating Averted Hospitalizations and Deaths Using Literature Estimates of Risk of Hospitalization and Death

eTable 8. Sensitivity Analysis Estimating Averted Hospitalizations and Deaths Using Estimates of Vaccine Effectiveness Against Hospitalization and Death

eReferences.

© 2022 Tan ST et al. *JAMA Network Open.*

This supplemental material has been provided by the authors to give readers additional information about their work.

© 2022 Tan ST et al. *JAMA Network Open.*

eAppendix. Supplemental Methods

In this appendix, we provide further methodologic detail on the model structure and statistical analysis.

Study outcomes

Calculation of relative reduction of COVID-19 cases

We estimated the relative reduction of COVID-19 cases in the entire vaccine-eligible population (≥12 years) and each age group (12-17 years, 18-49 years, 50-64 years, and ≥65 years) after the start of Phase 1A of vaccination (November 29, 2020), adjusting for vaccine coverage in the relevant population. The formula for percentage reduction in COVID-19 cases over a fixed period of time is as follows:

$$\%\ reduction\ in\ clinical\ cases = \frac{\frac{predicted\ cases - observed\ cases}{predicted\ cases}}{mean\ fraction\ vaccine\ coverage} \times 100$$

In sensitivity analysis, we estimated alternative formulations of the relative reduction: (1) accounting for age-specific eligibility over time; and (2) not adjusting by vaccine coverage (see Sensitivity analyses).

Estimation of averted COVID-19 cases: Additional methodology

Primary model

We defined the lower bound for the number of weekly averted COVID-19 cases as zero based on bioplausibility.

Alternative model

Model of natural immunity

We assumed that natural infection provided perfect immunity without waning though we relaxed this assumption in a sensitivity analysis. We assumed complete reporting of COVID-19 cases. We estimated total infections in the unvaccinated and each vaccine-eligible age group (<12 years, 12-17 years, 18-49 years, 50-64 years, ≥65 years) using literature estimates of the subclinical proportion by age (<19 years, 19-59 years, ≥60 years)[1]. We used the reported means and 95% confidence intervals of each age-specific subclinical fraction of infection to fit optimal beta distributions. The mean and the fitted shape parameters of each distribution are shown in Table A1.

© 2022 Tan ST et al. *JAMA Network Open.*

Table A1: Mean and fitted parameters for the distribution of subclinical fraction of SARS-CoV-2 infection by age group[1]

Age group (years)		Subclinical fraction	
Primary age groups	Age subgroups	Mean (95% CI)	Beta distribution (α, β)
<12	<12	0.47 (0.32, 0.62)	Beta (19.3, 21.8)
12-17	12-17	0.47 (0.32, 0.62)	Beta (19.3, 21.8)
18-49	18	0.47 (0.32, 0.62)	Beta (19.3, 21.8)
	19-49	0.32 (0.22, 0.44)	Beta (22.4, 46.7)
50-64	50-59	0.32 (0.22, 0.44)	Beta (22.4, 46.7)
	60-64	0.2 (0.13, 0.29)	Beta (17.6, 69.2)
≥65	≥65	0.2 (0.13, 0.29)	Beta (17.6, 69.2)

For each age group *a*, we estimated the total infections at week *t* using the following formula:

$$I_{a,t} = \sum_{i \epsilon a} \frac{C_{i,t}}{1 - p_i}$$

$I_{a,t} = \ total\ infections$
$C_{i,t} = observed\ COVID - 19\ cases$
$p_i = subclinical\ fraction$

Model of vaccine-induced immunity

We modeled vaccine effectiveness (against clinical disease) and waning immunity on a person-level based on vaccine (BNT162b2, mRNA-1273, Ad26.COV2.S) and the number of doses received. We assumed six possible vaccination scenarios: 1) BNT162b2 single dose; 2) BNT162b2 two doses; 3) mRNA-1273 single dose; 4) mRNA-1273 two doses; 5) Ad26.COV2.S single dose; and 6) unvaccinated. We did not include boosters given limited use over the study period.

We used published literature to estimate the vaccine effectiveness in each scenario over time, assuming instantaneous onset of protection and waning immunity at various time points[2–6]. We fit beta distributions using the published mean and 95% confidence intervals of each estimate of vaccine effectiveness (see Table A2). We made the simplifying assumption that all individuals who received two doses of the BNT162b2 vaccine received their second dose three weeks after their first dose and all individuals that received two doses of the mRNA-1273 vaccine received their second dose four weeks after receiving their first dose based on published literature[7]. We did not account for potential differences in vaccine effectiveness by age, which is broadly supported by literature[6,8,9]. We accounted for possible changes in vaccine effectiveness against the highly infectious Delta variant of SARS-CoV-2 as a sensitivity analysis (see Sensitivity analyses) but did not account for variant specific effectiveness in the main analysis. Average vaccine effectiveness and waning over time is shown in Figure A1, and the distributions of vaccine effectiveness are shown in Table A2.

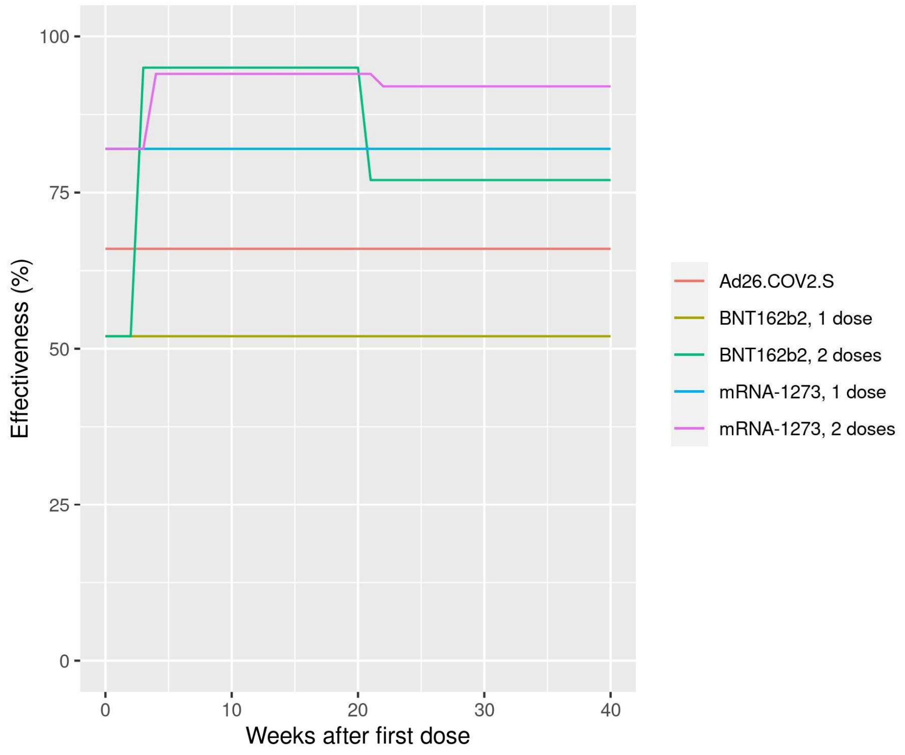

Figure A1: Mean COVID-19 vaccine effectiveness against clinical infection over time by vaccine and number of doses

Table A2: Mean and fitted parameters for COVID-19 vaccine effectiveness

		Vaccine effectiveness	
Vaccine	Vaccine period	Mean (95% CI)	Beta distribution (α, β)
BNT162b2	Between 1st and 2nd dose	0.52 (0.3, 0.68)[3]	Beta (12, 11.9)
	≤17 weeks after 2nd dose	0.95 (0.90, 0.98)[3]	Beta (128.5, 7.2)
	>17 weeks after 2nd dose	0.77 (0.67, 0.84)[5]	Beta (71.3, 22.1)
mRNA-1273	Between 1st and 2nd dose	0.82 (0.74, 0.87)[6]	Beta (109.1, 25.1)
	≤17 weeks after 2nd dose	0.94 (0.89, 0.97[2]	Beta (125.6, 8.4)
	>17 weeks after 2nd dose	0.92 (0.87, 0.96)[5]	Beta (125.3, 10.8)
Ad26.COV2.S	After 1st dose	0.66 (0.55, 0.75)[4]	Beta (55.7, 29.2)

We used publicly available COVID-19 vaccination data[10] to estimate the weekly number of newly vaccinated individuals in each of the six scenarios and age groups (12-17 years, 18-49 years, 50-64 years, ≥65 years). These age groups were based on vaccine prioritization age groupings. Date of receipt of first and second doses of the BNT162b2 and mRNA-1273 vaccines was not available in our data. We therefore calculated the mean fraction of individuals that received BNT162b2 or mRNA-1273 vaccines in each age group to estimate weekly BNT162b2 and mRNA-1273 vaccinations. We additionally used published literature to estimate the proportion of individuals who received only a single dose of the BNT162b2 or mRNA-1273 vaccines[7].

© 2022 Tan ST et al. *JAMA Network Open.*

We combined our estimates of the number of individuals newly vaccinated each week by vaccine type and number of doses received and corresponding vaccine effectiveness over time to estimate the fraction of the population with immunity due to vaccination. Since we assumed that natural infection provided perfect immunity, we first calculated the number of newly vaccinated individuals not previously infected with SARS-CoV-2 each week before estimating the number of protected individuals over time due to vaccination. We assumed previously infected and uninfected individuals were equally likely to receive any COVID-19 vaccine, following the observed weekly distribution of vaccines by vaccine type. The formula for calculating the number of new vaccinations in previously uninfected individuals in an age group a at week t is as follows:

$$v_{a,t} = V_{a,t}(1 - \frac{I_{a,t}}{N_{a,t}})$$

$v_{a,t} = \text{ new vaccinations in susceptible individuals}$
$V_{a,t} = \text{total new vaccinations}$
$I_{a,t} = \text{number of unvaccinated and previously infected persons}$
$N_{a,t} = \text{total unvaccinated persons}$

Monte Carlo simulation
We used Monte Carlo simulation to capture uncertainty for the analysis in the alternative model, with a focus on accounting for uncertainty in vaccine effectiveness and estimates of subclinical infection. We ran 1000 simulations using randomly sampled values of parameters from fitted parameter distributions (Table A1 and A2). We reported the mean and 95% uncertainty intervals (95% UI) of study outcomes.

To generate random samples of our parameters for each simulation, we independently sampled from the distributions of sub-clinical fractions in three age groups: <19 years, 19-59 years, and ≥60 years. We sampled independently from the standard uniform distribution for three vaccines (BNT162b2, mRNA-1273, and Ad26.COV2.S) and used inversion sampling to generate samples of vaccine effectiveness to account for changes in effectiveness over time.

<u>Estimation of averted COVID-19 hospitalizations and deaths: Additional methodology</u>
We estimated monthly risks of hospitalization and death in each age group of the population (<12 years, 12-17 years, 18-49 years, 50-64 years, ≥65 years) by finding the proportion of cases that resulted in hospitalization or death each month using CDPH data.

Due to lag in reporting of severe COVID-19 outcomes, we used the age-specific monthly risk of hospitalization and death in August 2021 to predict averted hospitalizations and deaths in September and October 2021 (eFigure 1). We defined the lower bound for the number of weekly averted COVID-19 hospitalizations and deaths as zero based on bioplausibility.

We also used values from literatures for risk of hospitalization and death from COVID-19 in sensitivity analysis.

<u>Prediction and uncertainty intervals</u>
Prediction intervals from the primary modeling approach reflect both uncertainty in the estimated model parameters and variation expected for the outcome, while the uncertainty intervals from

© 2022 Tan ST et al. *JAMA Network Open.*

the alternative modeling approach reflect the uncertainty in the parameters inputted into the alternative model. These represent different measures of statistical variability in estimation.

Sensitivity analyses

Age-specific vaccine eligibility

In both modeling approaches, we performed a sensitivity analysis to account for age-specific differences in COVID-19 vaccine eligibility over time among the four vaccine-eligible age groups (12-17 years, 18-49 years, 50-64 years, ≥65 years).

COVID-19 vaccines became available for the general population ≥16 years and 12-15 years in mid-April 2021 and mid-May 2021 respectively[11]. We assumed vaccination in the population 12-17 years began on April 11, 2021.

Vaccination in adults 18-49 years and 50-64 years began before vaccines were widely available in those populations due to occupational risk. Healthcare and other frontline workers became eligible for COVID-19 vaccines in Phase 1A of vaccination and essential workers became eligible for vaccines in Phase 1B of vaccination[11,12]. For this analysis, we assumed widespread vaccination in the populations 18-49 years and 50-64 years began with Phase 1B of vaccination and used February 14, 2021 to mark vaccine eligibility[13–16].

We used January 10, 2021 as the start of vaccination among adults ≥65 years, since they were eligible for COVID-19 vaccines beginning mid-January 2021[17].

We performed the main analysis in each age group after each age group became eligible for vaccines, reporting both unadjusted and adjusted relative reduction of outcomes. Dates of vaccine-eligibility by age group used in this analysis are shown in Table A3.

Table A3: Date of vaccine-eligibility by age group

Age group (years)	Date beginning vaccine-eligibility
12-17	April 11, 2021
18-49	February 14, 2021
50-64	February 14, 2021
≥65	January 10, 2021

Primary model

We evaluated the effects of varying the definition of onset of widespread vaccination from November 28, 2020 to January 2, 2021 in weekly timesteps.

We additionally examined the impact of using different age groups (12-17 years, <18 years) for the unvaccinated population. Incorporating some vaccine-eligible populations in the unvaccinated population reduced the estimated impact of vaccination.

The primary model relies on the assumption that COVID-19 cases in the unvaccinated population remain a reliable predictor of cases in the vaccine-eligible population in the absence of vaccination. This assumption may be challenged if there are age-based differences in risk of

© 2022 Tan ST et al. *JAMA Network Open.*

infection over time. We assessed the impact of changes in the risk of infection in the unvaccinated population due to the Delta variant. We conducted primary analysis under the scenarios where the unvaccinated population is at 10% higher and 10% lower risk of infection than the vaccine-eligible population after the spread of the Delta variant. We defined the introduction of the Delta variant as May 30, 2021.

Alternative model
Several variants of SARS-CoV-2 have been identified. Vaccines remained highly effective against infection among most SARS-CoV-2 variants[18,19], but there is evidence of decreased effectiveness of COVID-19 vaccines against symptomatic infection from the Delta variant of SARS-CoV-2[18,20,21]. We evaluated study outcomes when considering reduced effectiveness of vaccines against the Delta variant after June 2021. The distributions of vaccine effectiveness against clinical disease due to the Delta variant are below in Table A4. We did not perform this sensitivity analysis in the primary model since person-level vaccination was not explicitly included.

Table A4: Mean and fitted parameters for COVID-19 vaccine effectiveness against the SARS-CoV-2 Delta variant

		Vaccine effectiveness	
Vaccine	Vaccine period	Mean (95% CI)	Beta distribution (α, β)
BNT162b2	Between 1st and 2nd dose	0.52 (0.3, 0.68)[3,20]	Beta (12, 11.9)
	≤17 weeks after 2nd dose	0.95 (0.90, 0.98)[3,20]	Beta (128.5, 7.2)
	>17 weeks after 2nd dose	0.53 (0.39, 0.65)[20]	Beta (28.9, 26.3)
mRNA-1273	Between 1st and 2nd dose	0.77 (0.60, 0.87)[18]	Beta (30.2, 9.5)
	≤17 weeks after 2nd dose	0.94 (0.89, 0.97)[2,18]	Beta (125.6, 8.4)
	>17 weeks after 2nd dose	0.80 (0.70, 0.87)[18]	Beta (71.9, 18.7)
Ad26.COV2.S	After 1st dose	0.60 (0.31, 0.77)[21]	Beta (10, 7.8)

We conducted a separate sensitivity analysis that relaxed the assumption of perfect immunity from infection. We assumed that SARS-CoV-2 infection was 86% effective against reinfection within 1 year of primary infection with waning after 1 year based on recent published literature[22,23]. We included these estimates of effectiveness of natural infection against reinfection as additional parameters to sample from in the Monte Carlo simulation. The fitted beta distributions of these parameters are shown in Table A5. We additionally assumed that all COVID-19 vaccines were 90% effective against reinfection after previous natural infection, which is supported by literature[22].

Table A5: Mean and fitted parameters for effectiveness of previous COVID-19 infection against reinfection

Time since primary infection	Effectiveness against reinfection	
	Mean (95% CI)	Beta distribution (α, β)
≤1 year	0.86 (0.27, 0.97)[24]	Beta (1.7, 0.27)
>1 year	0.69 (0.38, 0.84)[24]	Beta (9.3, 5)

Hospitalizations and deaths

We assessed hospitalization and death outcomes of the main analyses when using literature estimates of the risk of hospitalization and death in cases that were not fully vaccinated[25] rather than estimates from CDPH data. A comparison of the hospitalization and death risk used in the main analyses and literature estimates are shown in Table A6.

Table A6: Estimates of hospitalization and death risk in vaccine era in the population ≥18 years

	Age group (years)	Estimated risk from CDPH data	Literature estimate of risk[25]
Risk of hospitalization (%)	18-49	2.1	3.2
	50-64	6.7	9.8
	≥65	19	21.5
Risk of death (%)	18-49	0.2	0.2
	50-64	1.7	1.5
	≥65	9.7	7.3

The alternative model we developed is applicable to other COVID-19 outcomes. As an additional sensitivity analysis, we adapted the alternative model to predict hospitalizations and deaths that would have occurred in the absence of vaccination. We estimated the incidence of hospitalization and deaths instead of incidence of cases, incorporating literature estimates of vaccine effectiveness against hospitalizations and death to estimate the susceptibility profile of the population. The distributions of vaccine effectiveness against hospitalization and death are in Table A7.

Table A7: Mean and fitted parameters for COVID-19 vaccine effectiveness against hospitalization and death

		Vaccine effectiveness against hospitalization		Vaccine effectiveness against death[26]	
Vaccine	Vaccine period	Mean (95% CI)	Beta distribution (α, β)	Mean (95% CI)	Beta distribution (α, β)
BNT162b2	Between 1st and 2nd dose	0.75 (0.21, 0.92)[20]	Beta (2.2, 0.8)	0.92 (0.89, 0.94)	Beta (400, 37.4)
	≤17 weeks after 2nd dose	0.95 (0.9, 0.98)[20]	Beta (105.3, 5.7)	0.98 (0.97, 0.99)	Beta (684.3, 12)
	>17 weeks after 2nd dose	0.77 (0.67, 0.84)[5]	Beta (71.3, 22.1)	0.92 (0.88, 0.95)	Beta (172.1, 14.8)
mRNA-1273	Between 1st and 2nd dose	0.82 (0.74, 0.87)[4]	Beta (30.2, 9.5)	0.88 (0.84, 0.92)	Beta (212.7, 28.8)
	≤17 weeks after 2nd dose	0.94 (0.89, 0.97)[2]	Beta (125.6, 8.4)	0.99 (0.97, 0.99)	Beta (521.8, 7.8)
	>17 weeks after 2nd dose	0.92 (0.87, 0.96)[5]	Beta (125.3, 10.8)	0.96 (0.92, 0.98)	Beta (179.8, 8.6)
Ad26.COV2.S	After 1st dose	0.84 (0.64, 0.93)[9]	Beta (19.1, 4.1)	0.82 (0.46, 0.94)	Beta (6, 1.5)
	>4 weeks after 1st dose	0.68 (0.49, 0.8)[5]	Beta (22.3, 11.3)	-	-

eFigure 1. Monthly Risk of Hospitalization and Death Among COVID-19 Cases

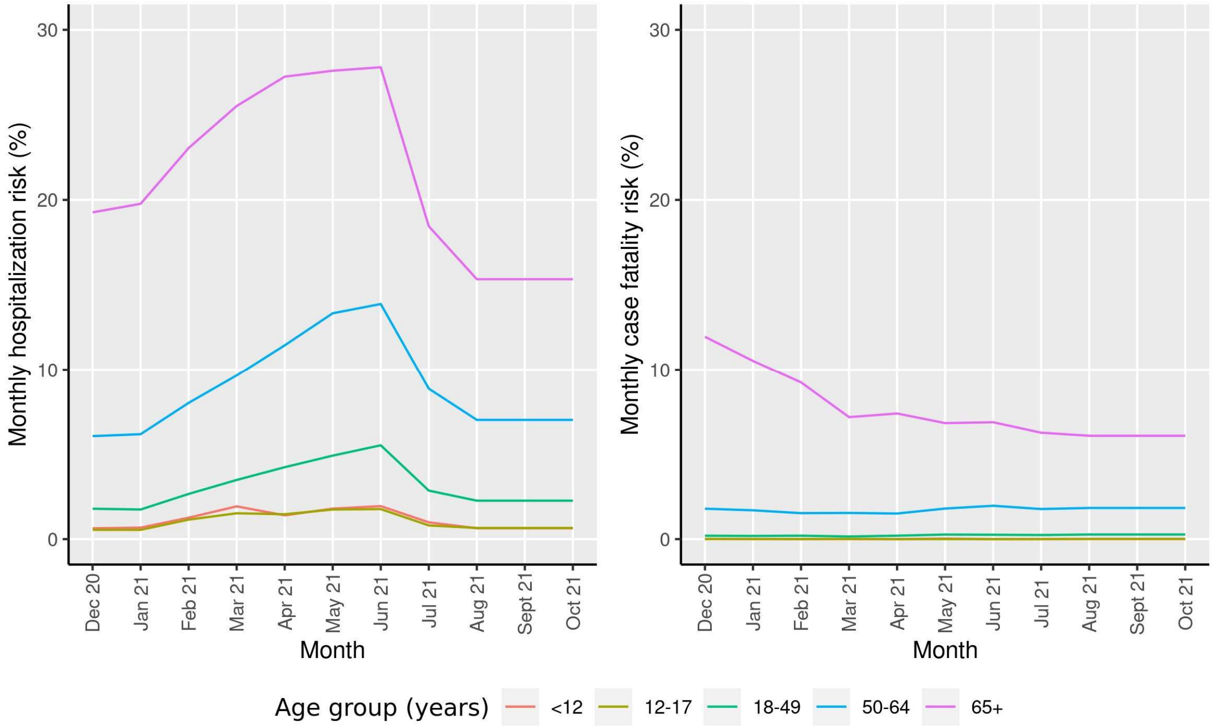

We plotted estimated probabilities of hospitalization (A) and deaths (B) among confirmed COVID-19 cases over time in the vaccine era (November 29, 2020 – October 16, 2021) in five age groups: (<12 years, 12-17 years, 18-49 years, 50-64 years, ≥65 years). Due to a lag in reporting of severe outcomes, we used the average risk of hospitalization and death in August 2021 as proxies for the risk of hospitalization and death in September and October of 2021. Literature values were comparable.

© 2022 Tan ST et al. *JAMA Network Open.*

eFigure 2. Model Calibration of Primary Model for Ratio Between COVID-19 Cases in the Unvaccinated and Vaccine-Eligible Age Groups in the Prevaccine Era

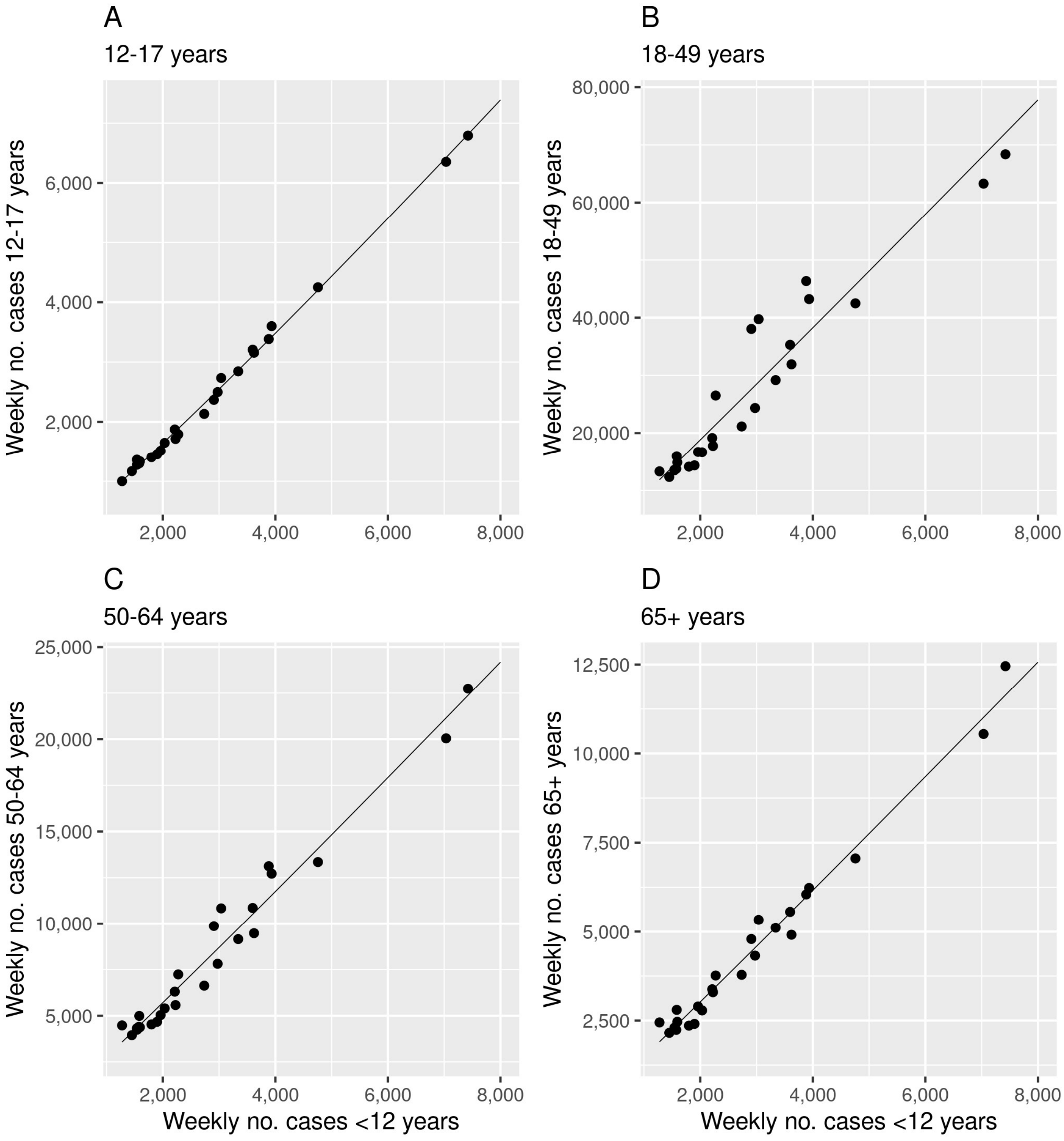

In our primary model, we plotted weekly cases in the unvaccinated population (<12 years) and each vaccine-eligible age group (12-17 years in panel A, 18-49 years in panel B, 50-64 years in panel C, and ≥65 years in panel D) before Phase 1a of vaccination. We fit quasi-Poisson models between log-transformed weekly cases in the unvaccinated population and weekly cases in each vaccine-eligible age group (black line) and observed good model fit in each age group. The strong linear relationship suggests a good model fit during the calibration. We used the calibrated models to make predictions on COVID-19 cases in the vaccine era under the scenario of no vaccination.

© 2022 Tan ST et al. *JAMA Network Open.*

eFigure 3. Comparison of Primary and Alternative Models of Estimating Vaccine-Averted COVID-19 Outcomes in the Population Aged 12 to 17 Years

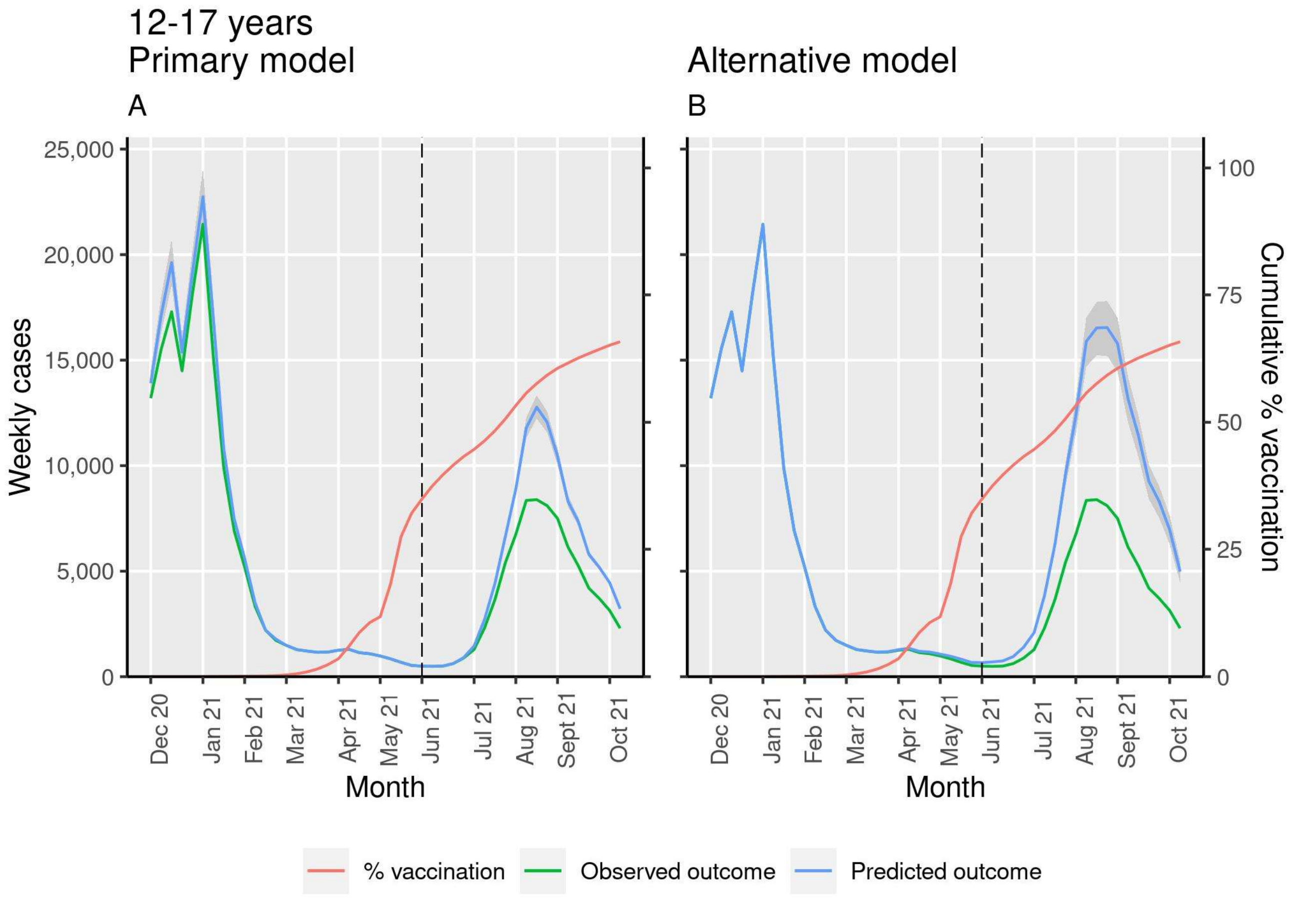

We compare the results estimating averted COVID-19 cases in the population 12-17 years from the primary model (A) and the alternative model (B). In both panels, we plot the vaccine coverage of at least 1 dose of a COVID-19 vaccine over the vaccine era (red). We plot the observed cases over time in green and the predicted cases in the absence of vaccination from each model in blue. The difference between the predicted cases in absence of vaccination and the observed cases represents averted cases due to COVID-19 vaccination. The dashed line represents the introduction of the Delta variant in California in June 2021 (black).

© 2022 Tan ST et al. *JAMA Network Open.*

eFigure 4. Comparison of Primary and Alternative Models of Estimating Vaccine-Averted COVID-19 Outcomes in the Population Aged 18 to 49 Years

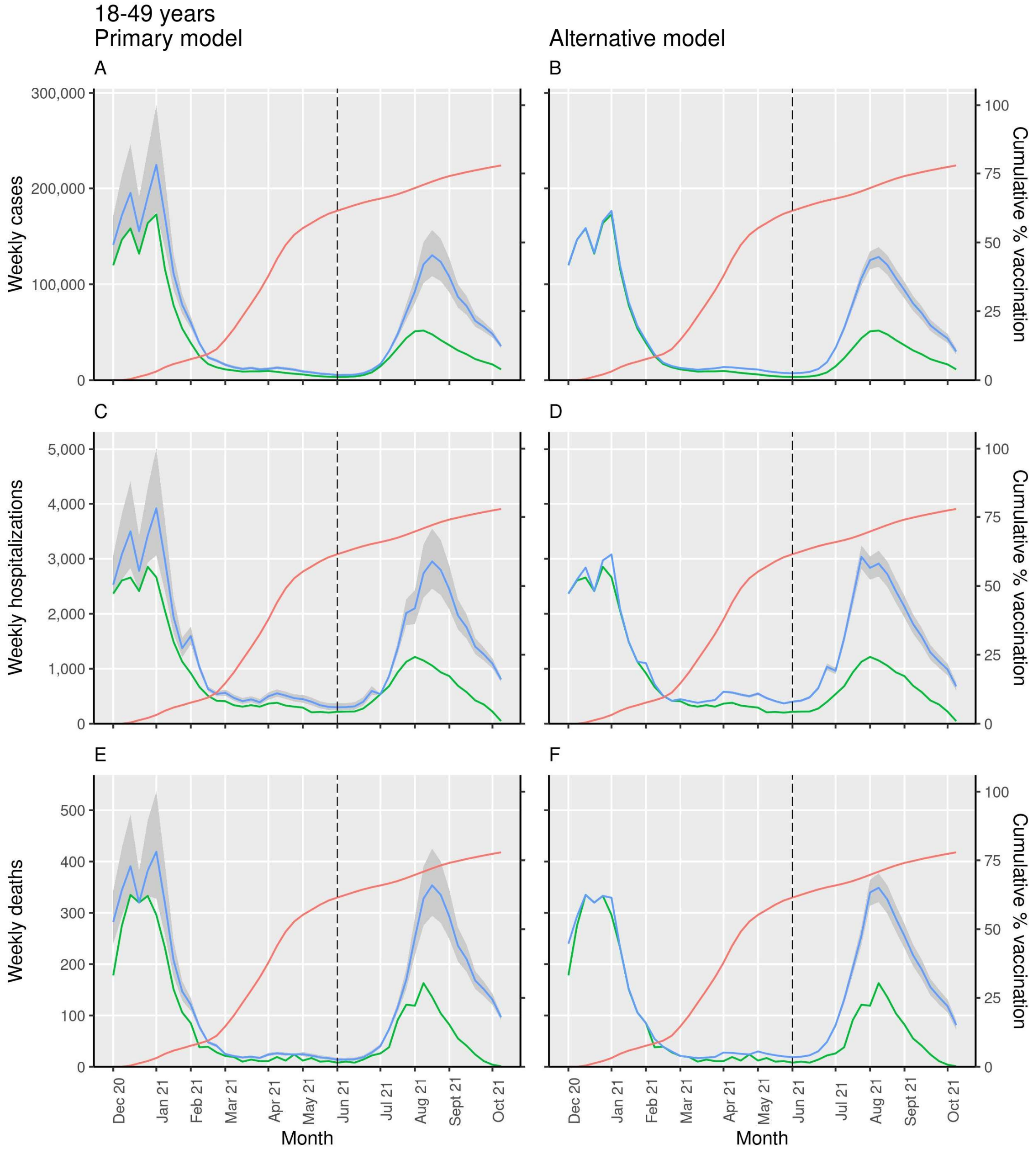

We compare the results estimating averted COVID-19 cases (A, B), hospitalizations (C, D), and deaths (E, F) in the population 18-49 years from the primary model (left) and the alternative model (right). In both panels, we plot the vaccine coverage of at least 1 dose of a COVID-19 vaccine over the vaccine era (red). We plot the observed outcome over time in green and the predicted outcome in the absence of vaccination from each model in blue. The difference between the predicted outcome in absence of vaccination and the observed outcome represents the averted outcome due to COVID-19 vaccination. The dashed line represents the introduction of the Delta variant in California in June 2021 (black).

© 2022 Tan ST et al. *JAMA Network Open.*

eFigure 5. Comparison of Primary and Alternative Models of Estimating Vaccine-Averted COVID-19 Outcomes in the Population Aged 50 to 64 Years

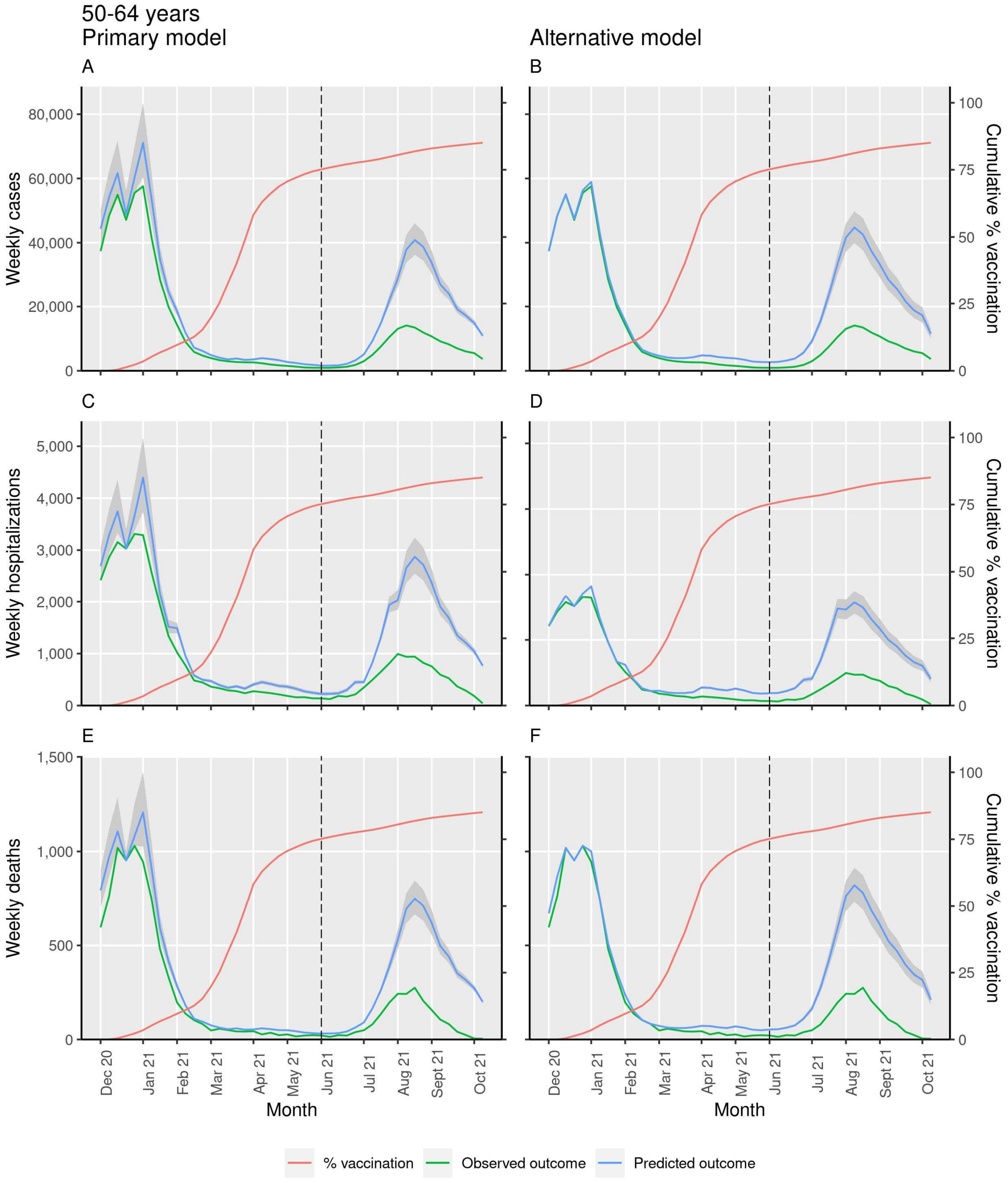

We compare the results estimating averted COVID-19 cases (A, B), hospitalizations (C, D), and deaths (E, F) in the population 50-64 years from the primary model (left) and the alternative model (right). In both panels, we plot the vaccine coverage of at least 1 dose of a COVID-19 vaccine over the vaccine era (red). We plot the observed outcome over time in green and the predicted outcome in the absence of vaccination from each model in blue. The difference between the predicted outcome in absence of vaccination and the observed outcome represents the averted outcome due to COVID-19 vaccination. The dashed line represents the introduction of the Delta variant in California in June 2021 (black).

© 2022 Tan ST et al. *JAMA Network Open.*

eFigure 6. Comparison of Primary and Alternative Models of Estimating Vaccine-Averted COVID-19 Outcomes in the Population Aged 65 Years or Older

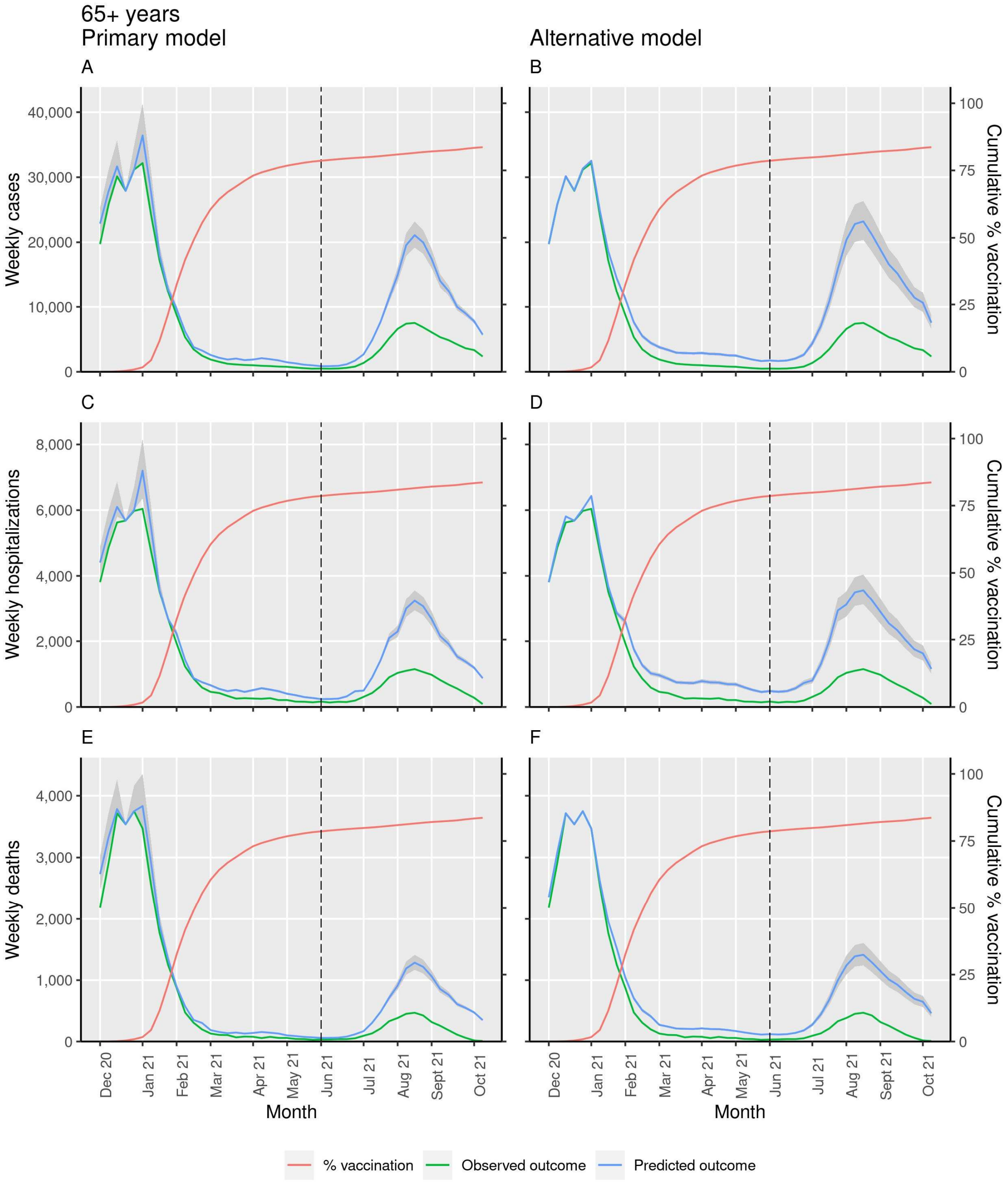

We compare the results estimating averted COVID-19 cases (A, B), hospitalizations (C, D), and deaths (E, F) in the population ≥65 years from the primary model (left) and the alternative model (right). In both panels, we plot the vaccine coverage of at least 1 dose of a COVID-19 vaccine over the vaccine era (red). We plot the observed outcome over time in green and the predicted outcome in the absence of vaccination from each model in blue. The difference between the predicted outcome in absence of vaccination and the observed outcome represents the averted outcome due to COVID-19 vaccination. The dashed line represents the introduction of the Delta variant in California in June 2021 (black).

© 2022 Tan ST et al. *JAMA Network Open.*

eTable 1. Sensitivity Analysis on Association of Age-Specific Vaccine Eligibility With Vaccine-Averted Outcomes in California

		Since Phase 1A			Since age-based eligibility*		
			Relative reduction in outcome (%) (95% PI or UI)**			Relative reduction in outcome (%) (95% PI or UI)**	
Outcome	Age group (years)	Averted COVID-19 outcome (95% PI or UI)	Unadjusted	Adjusted	Averted COVID-19 outcome (95% PI or UI)	Unadjusted	Adjusted
**COVID-19 case**							
Primary	≥12	1,523,500 (976,800, 2,230,800)	34 (25, 43)	72 (53, 91)	907,280 (804,840, 1,017,760)	46 (43, 49)	86 (81, 92)
	12-17	40,930 (30,300, 52,100)	15 (11, 18)	57 (44, 70)	24,340 (22,900, 25,850)	22 (21, 23)	51 (48, 53)
	18-49	1,036,700 (615,100, 1,588,400)	36 (25, 46)	83 (58, 100)	554,440 (479,460, 635,510)	47 (43, 50)	84 (78, 90)
	50-64	306,300 (221,500, 415,200)	34 (27, 41)	66 (52, 79)	206,160 (189,880, 223,310)	54 (52, 56)	81 (77, 83)
	≥65	139,500 (109,900, 180,200)	30 (25, 36)	49 (42, 59)	122,340 (112,600, 133,090)	43 (41, 45)	62 (59, 65)
Alternative	≥12	1,402,100 (1,192,100, 1,615,600)	32 (29, 35)	68 (61, 75)	1,052,140 (911,250, 1,184,090)	50 (47, 53)	93 (86, 99)
	12-17	78,760 (66,140, 90,610)	25 (22, 28)	97 (85, 100)	70,180 (59,590, 79,670)	45 (41, 48)	100 (94, 100)
	18-49	810,700 (697,990, 922,240)	31 (28, 34)	71 (64, 77)	592,820 (520,640, 658,400)	49 (45, 51)	87 (81, 92)
	50-64	321,280 (269,390, 375,390)	35 (31, 39)	68 (60, 75)	209,990 (182,690, 235,800)	55 (51, 57)	81 (76, 85)
	≥65	191,390 (158,570, 227,340)	37 (33, 41)	61 (54, 68)	179,170 (148,330, 210,220)	53 (48, 57)	76 (69, 82)
**COVID-19 hospitalization**							
Primary	≥18	72,930 (53,250, 99,150)	34 (27, 41)	70 (56, 84)	53,130 (47,730, 58,960)	47 (44, 49)	83 (78, 87)
	18-49	24,220 (15,520, 35,650)	38 (29, 48)	88 (66, 100)	14,670 (12,470, 17,060)	46 (42, 50)	83 (75, 89)
	50-64	23,300 (17,600, 30,620)	37 (31, 44)	71 (59, 84)	16,650 (15,240, 18,130)	55 (53, 57)	82 (78, 85)
	≥65	25,410 (20,140, 32,880)	29 (24, 35)	48 (40, 57)	21,810 (20,010, 23,760)	42 (40, 44)	60 (57, 63)
Alternative	≥18	84,330 (71,760, 97,510)	38 (34, 41)	76 (69, 83)	66,140 (56,790, 75,210)	52 (48, 55)	92 (86, 98)
	18-49	22,230 (19,390, 25,030)	36 (33, 39)	84 (77, 90)	16,080 (14,240, 17,760)	49 (45, 51)	87 (81, 91)
	50-64	26,650 (22,660, 30,790)	40 (37, 44)	77 (70, 84)	17,460 (15,340, 19,460)	56 (53, 59)	83 (79, 87)
	≥65	35,450 (29,710, 41,690)	36 (32, 40)	60 (53, 66)	32,590 (27,210, 37,990)	52 (47, 56)	75 (68, 80)
**COVID-19 death**							
Primary	≥18	19,430 (14,840, 26,230)	30 (25, 37)	61 (50, 75)	14,650 (13,510, 15,940)	48 (46, 50)	85 (81, 89)
	18-49	2,730 (1,880, 3,920)	43 (34, 52)	98 (78, 100)	1,670 (1,490, 1,870)	56 (53, 58)	99 (94, 100)
	50-64	6,070 (4,670, 7,920)	38 (32, 45)	73 (62, 86)	4,220 (3,930, 4,520)	62 (60, 63)	92 (89, 94)
	≥65	10,630 (8,290, 14,400)	25 (21, 31)	42 (34, 52)	8,760 (8,090, 9,560)	42 (40, 44)	61 (58, 64)
Alternative	≥18	22,620 (19,280, 26,190)	33 (30, 37)	68 (61, 75)	18,690 (16,020, 21,330)	54 (50, 57)	96 (89, 100)
	18-49	2,410 (2,130, 2,700)	40 (37, 42)	91 (84, 97)	1,790 (1,600, 1,960)	57 (54, 59)	100 (97, 100)
	50-64	6,490 (5,560, 7,460)	40 (36, 43)	76 (69, 83)	4,310 (3,820, 4,780)	62 (59, 65)	93 (88, 96)
	≥65	13,720 (11,600, 16,020)	30 (27, 34)	50 (44, 56)	12,590 (10,600, 14,590)	51 (47, 55)	74 (67, 79)

*Dates of vaccine eligibility varied by age group. We assumed vaccine eligibility began for 12-17 years on April 11, 2021, 18-49 years on February 14, 2021, 50-64 years on February 14, 2021, and ≥65 years on January 10, 2021.

**Relative reduction in outcomes were adjusted for the mean vaccine coverage in the population during the vaccine era, while the unadjusted estimate did not account for vaccine coverage

© 2022 Tan ST et al. *JAMA Network Open.*

eTable 2. Sensitivity Analysis of the Primary Model Measuring Averted COVID-19 Outcomes When Varying the Start of the Vaccine Era in California

Outcome	Start of vaccination	Averted COVID-19 outcome (95% PI)	Relative reduction in outcome (%) (95% PI)*	
			Unadjusted	Adjusted
COVID-19 case	November 29, 2020	1,523,500 (976,800, 2,230,800)	34 (25, 43)	72 (53, 91)
	December 6, 2020	1,194,900 (925,110, 1,574,770)	30 (25, 36)	62 (52, 75)
	December 13, 2020	1,151,740 (950,400, 1,408,130)	31 (27, 36)	63 (55, 72)
	December 20, 2020	1,113,950 (957,140, 1,303,570)	33 (29, 36)	65 (59, 72)
	December 27, 2020	1,122,320 (974,760, 1,290,440)	35 (32, 38)	68 (62, 75)
	January 3, 2021	1,129,500 (994,120, 1,272,920)	38 (36, 41)	73 (67, 79)
COVID-19 hospitalization	November 29, 2020	72,930 (53,250, 99,150)	34 (27, 41)	70 (56, 84)
	December 6, 2020	60,850 (51,060, 74,750)	32 (28, 36)	63 (56, 72)
	December 13, 2020	60,300 (52,410, 70,080)	33 (30, 37)	65 (59, 71)
	December 20, 2020	59,550 (52,810, 67,230)	35 (32, 38)	67 (62, 72)
	December 27, 2020	60,910 (54,020, 68,590)	38 (35, 41)	71 (66, 76)
	January 3, 2021	61,130 (54,760, 67,980)	41 (39, 44)	75 (70, 80)
COVID-19 death	November 29, 2020	19,430 (14,840, 26,230)	30 (25, 37)	61 (50, 75)
	December 6, 2020	15,490 (13,640, 18,400)	27 (24, 30)	54 (49, 61)
	December 13, 2020	15,240 (13,730, 17,220)	29 (26, 31)	56 (51, 61)
	December 20, 2020	15,380 (14,030, 17,010)	32 (30, 34)	60 (57, 65)
	December 27, 2020	16,020 (14,410, 17,800)	36 (34, 39)	67 (63, 72)
	January 3, 2021	16,190 (14,690, 17,800)	41 (39, 43)	75 (70, 79)

*Relative reduction in outcomes were adjusted for the mean vaccine coverage in the population during the vaccine era, while the unadjusted estimate did not account for vaccine coverage

© 2022 Tan ST et al. *JAMA Network Open.*

eTable 3. Sensitivity Analysis of the Primary Model Measuring Averted COVID-19 Outcomes in Vaccine-Eligible Population Using Different Unvaccinated Age Groups

Outcome	Unvaccinated population (years)	Vaccine-eligible population (years)	Averted COVID-19 outcome (95% PI)	Relative reduction in outcome (%) (95% PI)*	
				Unadjusted	Adjusted
COVID-19 case	<18	≥18	1,213,660 (775,470, 1,816,300)	31 (22, 40)	62 (45, 81)
	12-17	≥18	936,650 (594,770, 1,457,450)	25 (18, 35)	52 (36, 71)
COVID-19 hospitalization	<18	≥18	61,010 (45,460, 83,250)	30 (24, 37)	62 (50, 76)
	12-17	≥18	49,180 (37,390, 67,800)	26 (21, 33)	53 (43, 66)
COVID-19 death	<18	≥18	15,990 (12,620, 21,560)	26 (22, 32)	53 (45, 66)
	12-17	≥18	12,710 (10,310, 17,010)	22 (19, 27)	45 (38, 56)

The base case analysis uses <12 as the unvaccinated population, this sensitivity analysis uses <18 and 12-17 years as alternative definitions.

*Relative reduction in outcomes were adjusted for the mean vaccine coverage in the population during the vaccine era, while the unadjusted estimate did not account for vaccine coverage

© 2022 Tan ST et al. *JAMA Network Open.*

eTable 4. Sensitivity Analysis of the Primary Model Measuring Changing Risk of Infection From the Delta Variant in Unvaccinated Population

Outcome	Age group (years)	Greater infectiousness of Delta variant in young children: Averted COVID-19 outcome (95% PI)	Greater infectiousness: Relative reduction in outcome (%) (95% PI)* Unadjusted	Greater infectiousness: Relative reduction in outcome (%) (95% PI)* Adjusted	Reduced infectiousness of Delta variant in young children: Averted COVID-19 outcome (95% PI)	Reduced infectiousness: Relative reduction in outcome (%) (95% PI)* Unadjusted	Reduced infectiousness: Relative reduction in outcome (%) (95% PI)* Adjusted
COVID-19 case	≥12	1,339,650 (825,160, 2,009,530)	31 (22, 40)	66 (46, 86)	1,708,000 (1,128,170, 2,455,490)	36 (27, 45)	78 (58, 96)
	12-17	29,500 (19,480, 40,010)	11 (8, 14)	43 (29, 56)	52,580 (41,250, 64,490)	18 (15, 21)	70 (57, 83)
	18-49	920,080 (522,920, 1,437,910)	33 (22, 44)	77 (51, 100)	1,153,650 (707,120, 1,731,070)	39 (28, 49)	89 (64, 100)
	50-64	269,510 (189,690, 372,580)	31 (24, 39)	60 (47, 74)	343,340 (253,130, 458,300)	37 (30, 44)	71 (58, 84)
	≥65	120,560 (93,070, 159,020)	27 (22, 33)	45 (37, 54)	158,430 (126,670, 201,640)	33 (28, 38)	54 (46, 63)
COVID-19 hospitalization	≥18	64,310 (45,890, 89,070)	31 (25, 39)	64 (50, 79)	81,580 (60,580, 109,390)	40 (30, 44)	81 (61, 89)
	18-49	21,410 (13,280, 32,170)	36 (25, 45)	82 (59, 100)	27,040 (17,750, 39,230)	45 (31, 50)	100 (72, 100)
	50-64	20,540 (15,210, 27,440)	34 (28, 41)	66 (53, 79)	26,070 (19,980, 33,840)	44 (34, 46)	83 (64, 89)
	≥65	22,360 (17,410, 29,460)	26 (22, 32)	44 (36, 53)	28,480 (22,850, 36,330)	34 (27, 37)	56 (44, 61)
COVID-19 death	≥18	17,290 (12,980, 23,760)	28 (22, 35)	56 (46, 70)	21,590 (16,700, 28,730)	35 (27, 39)	70 (55, 79)
	18-49	2,420 (1,630, 3,530)	40 (31, 49)	91 (71, 100)	3,050 (2,120, 4,310)	50 (37, 54)	100 (84, 100)
	50-64	5,400 (4,090, 7,140)	36 (29, 42)	68 (56, 81)	6,750 (5,250, 8,710)	44 (35, 47)	85 (67, 90)
	≥65	9,470 (7,260, 13,090)	23 (19, 29)	38 (31, 48)	11,790 (9,320, 15,710)	29 (23, 33)	47 (38, 55)

*Relative reduction in outcomes were adjusted for the mean vaccine coverage in the population during the vaccine era, while the unadjusted estimate did not account for vaccine coverage

© 2022 Tan ST et al. *JAMA Network Open.*

eTable 5. Sensitivity Analysis of the Alternative Modeling Adjusting for Possible Reduced Vaccine Effectiveness Against the SARS-CoV-2 Delta Variant

Outcome	Age group (years)	Averted COVID-19 outcome (95% UI)	Relative reduction in outcome (%) (95% UI)*	
			Unadjusted	Adjusted
COVID-19 case	≥12	1,106,300 (913,300, 1,308,650)	27 (23, 30)	58 (50, 65)
	12-17	75,190 (62,680, 86,880)	24 (21, 27)	93 (81, 100)
	18-49	657,090 (548,600, 768,420)	26 (23, 30)	61 (53, 68)
	50-64	241,790 (196,700, 291,550)	29 (25, 33)	56 (48, 63)
	≥65	132,190 (105,340, 161,810)	29 (24, 33)	48 (40, 55)
COVID-19 hospitalization	≥18	65,440 (54,350, 77,350)	32 (28, 36)	65 (57, 72)
	18-49	18,580 (15,820, 21,390)	32 (29, 35)	74 (67, 82)
	50-64	20,840 (17,320, 24,700)	35 (31, 39)	66 (59, 74)
	≥65	26,020 (21,210, 31,250)	29 (25, 33)	49 (42, 55)
COVID-19 death	≥18	17,120 (14,290, 20,220)	28 (24, 31)	56 (49, 63)
	18-49	2,000 (1,730, 2,290)	35 (32, 38)	81 (73, 88)
	50-64	5,030 (4,230, 5,930)	34 (30, 38)	65 (58, 72)
	≥65	10,090 (8,340, 12,010)	24 (21, 28)	40 (35, 46)

*Relative reduction in outcomes were adjusted for the mean vaccine coverage in the population during the vaccine era, while the unadjusted estimate did not account for vaccine coverage

We incorporated estimates of vaccine effectiveness against the Delta variant after June 2021 (see Table A4 for literature estimates) in the main analysis.

© 2022 Tan ST et al. *JAMA Network Open.*

eTable 6. Sensitivity Analysis of the Alternative Modeling With Waning Natural Immunity

Outcome	Age group (years)	Averted COVID-19 outcome (95% UI)	Relative reduction in outcome (%) (95% UI)*	
			Unadjusted	Adjusted
COVID-19 case	≥12	1,385,440 (1,182,580, 1,592,560)	32 (28, 35)	68 (60, 74)
	12-17	77,970 (65,910, 89,300)	25 (22, 27)	96 (84, 100)
	18-49	799,420 (691,520, 908,190)	30 (27, 33)	70 (63, 76)
	50-64	317,760 (267,470, 370,090)	35 (31, 39)	67 (60, 74)
	≥65	190,290 (157,680, 224,980)	37 (33, 41)	61 (54, 67)
COVID-19 hospitalization	≥18	83,560 (71,270, 96,320)	37 (34, 41)	76 (68, 83)
	18-49	21,940 (19,210, 24,670)	36 (33, 39)	83 (76, 89)
	50-64	26,370 (22,500, 30,380)	40 (36, 44)	77 (70, 83)
	≥65	35,250 (29,550, 41,270)	36 (32, 40)	60 (53, 66)
COVID-19 death	≥18	22,460 (19,180, 25,900)	33 (30, 37)	68 (61, 74)
	18-49	2,380 (2,110, 2,670)	39 (36, 42)	90 (84, 97)
	50-64	6,420 (5,520, 7,370)	40 (36, 43)	76 (69, 82)
	≥65	13,650 (11,540, 15,870)	30 (27, 33)	50 (44, 55)

*Relative reduction in outcomes were adjusted for the mean vaccine coverage in the population during the vaccine era, while the unadjusted estimate did not account for vaccine coverage

© 2022 Tan ST et al. *JAMA Network Open.*

eTable 7. Sensitivity Analysis Estimating Averted Hospitalizations and Deaths Using Literature Estimates of Risk of Hospitalization and Death

Outcome	Age group (years)	Averted COVID-19 outcome (95% PI or UI)	Relative reduction in outcome (%) (95% PI or UI)*	
			Unadjusted	Adjusted
**COVID-19 hospitalization**				
Primary	≥18	138,700 (111,140, 175,200)	50 (44, 56)	100 (90, 100)
	18-49	52,470 (39,480, 69,610)	57 (50, 64)	100 (100, 100)
	50-64	48,350 (40,060, 59,000)	55 (50, 60)	100 (97, 100)
	≥65	37,880 (31,600, 46,590)	38 (34, 43)	63 (56, 71)
Alternative	≥18	143,820 (128,110, 160,370)	51 (48, 53)	100 (97, 100)
	18-49	45,040 (41,470, 48,590)	54 (52, 56)	100 (100, 100)
	50-64	49,800 (44,710, 55,100)	56 (53, 58)	100 (100, 100)
	≥65	48,970 (41,930, 56,690)	44 (40, 48)	73 (67, 79)
**COVID-19 death**				
Primary	≥18	16,140 (13,590, 19,510)	26 (23, 30)	54 (47, 61)
	18-49	1,630 (1,010, 2,590)	31 (21, 41)	71 (49, 95)
	50-64	3,890 (3,040, 5,140)	28 (24, 34)	54 (45, 66)
	≥65	10,620 (9,550, 11,770)	25 (23, 27)	42 (38, 45)
Alternative	≥18	21,020 (17,760, 24,540)	32 (28, 35)	65 (58, 72)
	18-49	1,350 (1,150, 1,550)	27 (24, 30)	62 (55, 68)
	50-64	4,630 (3,880, 5,410)	32 (28, 36)	61 (54, 68)
	≥65	15,040 (12,730, 17,570)	32 (29, 36)	53 (47, 59)

*Relative reduction in outcomes were adjusted for the mean vaccine coverage in the population during the vaccine era, while the unadjusted estimate did not account for vaccine coverage
Literature estimates of hospitalization and death risks by age are shown in Table A6.

© 2022 Tan ST et al. *JAMA Network Open.*

eTable 8. Sensitivity Analysis Estimating Averted Hospitalizations and Deaths Using Estimates of Vaccine Effectiveness Against Hospitalization and Death

Outcome	Age group (years)	Averted COVID-19 outcome (95% UI)	Relative reduction in outcome (%) (95% UI)*	
			Unadjusted	Adjusted
COVID-19 hospitalization	≥18	81,700 (64,000, 96,800)	37 (31, 41)	75 (64, 83)
	18-49	21,540 (17,260, 24,860)	36 (31, 39)	82 (71, 90)
	50-64	25,320 (19,880, 30,100)	39 (34, 43)	75 (64, 83)
	≥65	34,830 (26,900, 41,860)	36 (30, 40)	59 (50, 66)
COVID-19 death	≥18	24,700 (22,200, 26,900)	35 (33, 37)	72 (67, 76)
	18-49	2,340 (2,140, 2,500)	39 (37, 40)	89 (84, 93)
	50-64	6,890 (5,990, 7,650)	41 (38, 44)	79 (73, 84)
	≥65	15,440 (14,060, 16,730)	33 (31, 35)	54 (51, 57)

*Relative reduction in outcomes were adjusted for the mean vaccine coverage in the population during the vaccine era, while the unadjusted estimate did not account for vaccine coverage

We adapted the alternative model that estimated vaccine-averted cases and incorporated estimates of vaccine effectiveness against hospitalization and death (see Table A7 for literature estimates).

© 2022 Tan ST et al. *JAMA Network Open.*

eReferences

1. Sah P, Fitzpatrick MC, Zimmer CF, et al. Asymptomatic SARS-CoV-2 infection: A systematic review and meta-analysis. *Proc Natl Acad Sci*. 2021;118(34). doi:10.1073/pnas.2109229118

2. Baden LR, El Sahly HM, Essink B, et al. Efficacy and Safety of the mRNA-1273 SARS-CoV-2 Vaccine. *N Engl J Med*. 2021;384(5):403-416. doi:10.1056/NEJMoa2035389

3. Polack FP, Thomas SJ, Kitchin N, et al. Safety and Efficacy of the BNT162b2 mRNA Covid-19 Vaccine. *N Engl J Med*. 2020;383(27):2603-2615. doi:10.1056/NEJMoa2034577

4. Sadoff J, Gray G, Vandebosch A, et al. Safety and Efficacy of Single-Dose Ad26.COV2.S Vaccine against Covid-19. *N Engl J Med*. 2021;384(23):2187-2201. doi:10.1056/NEJMoa2101544

5. Self WH. Comparative Effectiveness of Moderna, Pfizer-BioNTech, and Janssen (Johnson & Johnson) Vaccines in Preventing COVID-19 Hospitalizations Among Adults Without Immunocompromising Conditions — United States, March–August 2021. *MMWR Morb Mortal Wkly Rep*. 2021;70. doi:10.15585/mmwr.mm7038e1

6. Pilishvili T. Interim Estimates of Vaccine Effectiveness of Pfizer-BioNTech and Moderna COVID-19 Vaccines Among Health Care Personnel — 33 U.S. Sites, January–March 2021. *MMWR Morb Mortal Wkly Rep*. 2021;70. doi:10.15585/mmwr.mm7020e2

7. Kriss JL. COVID-19 Vaccine Second-Dose Completion and Interval Between First and Second Doses Among Vaccinated Persons — United States, December 14, 2020−February 14, 2021. *MMWR Morb Mortal Wkly Rep*. 2021;70. doi:10.15585/mmwr.mm7011e2

8. Olson SM. Effectiveness of Pfizer-BioNTech mRNA Vaccination Against COVID-19 Hospitalization Among Persons Aged 12–18 Years — United States, June–September 2021. *MMWR Morb Mortal Wkly Rep*. 2021;70. doi:10.15585/mmwr.mm7042e1

9. Moline HL. Effectiveness of COVID-19 Vaccines in Preventing Hospitalization Among Adults Aged ≥65 Years — COVID-NET, 13 States, February–April 2021. *MMWR Morb Mortal Wkly Rep*. 2021;70. doi:10.15585/mmwr.mm7032e3

10. COVID-19 Vaccine Progress Dashboard Data - COVID-19 Vaccines Administered By Demographics - California Health and Human Services Open Data Portal. Accessed November 17, 2021. https://data.chhs.ca.gov/dataset/vaccine-progress-dashboard/resource/faee36da-bd8c-40f7-96d4-d8f283a12b0a

11. Updated COVID-19 Vaccine Eligibility Guidelines. Accessed December 2, 2021. https://www.cdph.ca.gov/Programs/CID/DCDC/Pages/COVID-19/VaccineAllocationGuidelines.aspx

© 2022 Tan ST et al. *JAMA Network Open.*

12. Dooling K. The Advisory Committee on Immunization Practices' Updated Interim Recommendation for Allocation of COVID-19 Vaccine — United States, December 2020. *MMWR Morb Mortal Wkly Rep*. 2021;69. doi:10.15585/mmwr.mm695152e2

13. San Francisco Moves to Phase 1B of COVID-19 Vaccinations, Expands Eligibility to Educators, Child Care, Emergency Services, Food and Agriculture Workers | Office of the Mayor. Accessed December 5, 2021. https://sfmayor.org/article/san-francisco-moves-phase-1b-covid-19-vaccinations-expands-eligibility-educators-child-care

14. County A. Alameda County Gets a Mega Vaccination Site through State-Federal Partnership. :3.

15. Contra Costa Extends COVID-19 Vaccine Eligibility to Essential Workers :: Press Releases :: Contra Costa Health Services. Accessed December 5, 2021. https://cchealth.org/press-releases/2021/0218-COVID-19-Vaccine-Eligibility-to-Essential-Workers.php

16. Phases and Eligibility. Accessed December 5, 2021. https://www.sandiegocounty.gov/content/sdc/hhsa/programs/phs/community_epidemiology/dc/2019-nCoV/vaccines/phases.html

17. Seniors 65+ Now Eligible to Receive COVID-19 Vaccine to Effectively and Efficiently Increase Vaccine Distribution, Reduce Hospitalizations and Save Lives. Accessed December 2, 2021. https://www.cdph.ca.gov/Programs/OPA/Pages/NR21-015.aspx

18. Bruxvoort KJ, Sy LS, Qian L, et al. Effectiveness of mRNA-1273 against delta, mu, and other emerging variants of SARS-CoV-2: test negative case-control study. *BMJ*. 2021;375:e068848. doi:10.1136/bmj-2021-068848

19. Abu-Raddad LJ, Chemaitelly H, Butt AA. Effectiveness of the BNT162b2 Covid-19 Vaccine against the B.1.1.7 and B.1.351 Variants. *N Engl J Med*. 2021;385(2):187-189. doi:10.1056/NEJMc2104974

20. Tartof SY, Slezak JM, Fischer H, et al. Effectiveness of mRNA BNT162b2 COVID-19 vaccine up to 6 months in a large integrated health system in the USA: a retrospective cohort study. *The Lancet*. 2021;398(10309):1407-1416. doi:10.1016/S0140-6736(21)02183-8

21. Grannis SJ. Interim Estimates of COVID-19 Vaccine Effectiveness Against COVID-19–Associated Emergency Department or Urgent Care Clinic Encounters and Hospitalizations Among Adults During SARS-CoV-2 B.1.617.2 (Delta) Variant Predominance — Nine States, June–August 2021. *MMWR Morb Mortal Wkly Rep*. 2021;70. doi:10.15585/mmwr.mm7037e2

22. Hall V, Foulkes S, Insalata F, et al. Protection against SARS-CoV-2 after Covid-19 Vaccination and Previous Infection. *N Engl J Med*. 2022;0(0):null. doi:10.1056/NEJMoa2118691

© 2022 Tan ST et al. *JAMA Network Open.*

23. Chemaitelly H, Bertollini R, Abu-Raddad LJ. Efficacy of Natural Immunity against SARS-CoV-2 Reinfection with the Beta Variant. *N Engl J Med*. 2021;385(27):2585-2586. doi:10.1056/NEJMc2110300

24. Andrews N, Tessier E, Stowe J, et al. Duration of Protection against Mild and Severe Disease by Covid-19 Vaccines. *N Engl J Med*. 2022;0(0):null. doi:10.1056/NEJMoa2115481

25. Scobie HM. Monitoring Incidence of COVID-19 Cases, Hospitalizations, and Deaths, by Vaccination Status — 13 U.S. Jurisdictions, April 4–July 17, 2021. *MMWR Morb Mortal Wkly Rep*. 2021;70. doi:10.15585/mmwr.mm7037e1

26. Lin DY, Gu Y, Wheeler B, et al. Effectiveness of Covid-19 Vaccines over a 9-Month Period in North Carolina. *N Engl J Med*. 2022;0(0):null. doi:10.1056/NEJMoa2117128

27. Husereau D, Drummond M, Augustovski F, et al. Consolidated Health Economic Evaluation Reporting Standards 2022 (CHEERS 2022) statement: Updated reporting guidance for health economic evaluations. *Health Policy OPEN*. 2022;3:100063. doi:10.1016/j.hpopen.2021.100063

© 2022 Tan ST et al. *JAMA Network Open.*
